# The longitudinal relations between mental state talk and theory of mind

**DOI:** 10.1186/s40359-024-01692-y

**Published:** 2024-04-06

**Authors:** Isac Sehlstedt, Isabelle Hansson, Erland Hjelmquist

**Affiliations:** Department of Psychology, University of Gothenburg, P.O. Box 500, Gothenburg, SE 405 30 Sweden

**Keywords:** Theory of mind, Mental state talk, Executive function, Language, Social factors

## Abstract

**Background:**

Previous investigations of associations between children’s Theory of Mind (ToM) and parents’ use of words relating to mental states (or mental state talk; MST) have predominantly been performed using cross-sectional designs and false belief tasks as indicators of ToM.

**Methods:**

We here report a longitudinal study of 3–5 year-olds (*n* = 80) investigating ToM development using the ToM scale and three different parental MST types: the absolute frequency of words, the proportions of words, and the vocabulary size.

**Results:**

Our results revealed significant relations between all parental MST types and later child ToM. Proportions of parental MST were most often related to the children’s ToM at 4 years of age. However, the rate at which the children developed ToM from 3 to 5 years of age was associated with the other two parental MST type measures, namely, absolute frequency and vocabulary size. Additionally, our analyses revealed that parents’ use of cognitive MST words (e.g., think, or know) were most frequently associated with children’s ToM at 4 years of age compared to emotion and desire-related MST words.

**Conclusions:**

We conclude that the parental ability to capture the thoughts, beliefs, and knowledge present in different scenarios is associated with children’s ability to understand other minds. Moreover, parents’ way of talking about the mental states of others is associated with their children’s ability to understand and further develop ToM.

**Supplementary Information:**

The online version contains supplementary material available at 10.1186/s40359-024-01692-y.

Research regarding the development of social cognition has been of great interest for decades. One defining moment in this endeavor is Premack and Woordruff’s [1] application of the concept theory of mind (ToM), i.e., the ability to infer and understand one’s own and others’ beliefs, desires, knowledge, and intentions (theory of mind; ToM). The so-called litmus test of ToM ability focuses on children’s ability to understand false belief (FB), and many different FB tests have become widely used [2, 3]. An example of FB is when a child is presented with a scenario where a ball is placed in a basket by a teddy bear who subsequently leaves the basket unattended. Then, a doll removes the ball from the basket and puts it in a box. The child is then asked to guess where the teddy bear will look for the ball when the teddy bear comes back. If the child realizes that the teddy bear will look in the basket, the child understands FB (after [4, 5]).

There are now complementary methods for measuring ToM that does not exclusively test FB [6, 7], such as the ToM scale developed by Wellman and Liu [8]. This test captures a stagewise increase in ToM understanding in young children. Also, the scale can be used with a variable number of steps depending on the age group being tested. For example, the typical number of steps tested in preschool years is 4 or 5. However, the steps are not always found to have the same difficulty order, e.g., between countries [9] or even within a particular country [10]. Moreover, the 4-step version of the ToM scale seems reliable overall, but not its 5-step version [9]. For example, many studies have reported that the 5-step scale was unreliable, whereas a 4-step scale was [11–14]. The first four steps of the scale thus seem stable, but not without cultural differences.

The current study investigates the relationship between ToM and parents’ use of specific words, namely mental state talk (MST). MST can be separated into three categories which refer to words relating to (1) cognitions (e.g., believe, think, know), (2) emotions (e.g., happy, sad, angry), or (3) desires (e.g., want, like). Parental MST positively predicts children’s ToM ability [15, 16], especially between the use of cognition words and FB understanding [16–18]. In general, Devine and Hughes [15] reported that MST predicts false belief, FB; however, the strength of the relationships varied, and effect sizes were sometimes relatively small, albeit consistent.

Carr et al. [19] could not find a predictive relationship between mothers’ total early MST (at 3–4 years of age) and children’s later ToM (at 10 years of age), when controlling for earlier language and ToM levels. This result contradicts the finding of Devine and Hughes [20] and the meta-analysis by Devine and Hughes [15] where positive relationships between MST and later FB were found.

In Devine and Hughes [20] cognition terms accounted for about 60% of all mental state terms, with desire and emotion approximately an equal share of the remaining 40%, making it probable that cognition terms in particular account for the relation to FB. Direct support for a relation between parental cognitive MST and children’s later FB comes from Ensor and Hughes [21]; early ages, and Ensor et al. [18] later ages. Tompkins et al. [16] in a meta-analysis found empirical support for the positive effect of cognitive mental state terms as compared to desire and emotion mental state terms for infant/toddlers but not for preschool children. Altogether, there is theoretical and empirical ground for hypothesizing a difference between the three mental state categories, to the effect that cognitive mental states will have a more positive effect on ToM compared to desire and emotion terms.

The absolute frequency and the proportion of parental MST (i.e., the number of spoken words within each MST category divided by the total number of words uttered) have been used in previous research. However, few studies report both measures, absolute frequency being the most common [16]. The preponderance of absolute frequency measures might be explained by the hypothesis that ToM is positively affected each time a child hears a MST word, i.e., ToM is partly dependent on the frequency of parental MST input [17, 22, 23]. However, the strength of the measure of the proportion is that potentially irrelevant factors (e.g., talking time, number of uttered words) are controlled for [24]. Studies sometimes report absolute and proportional measures, and the results are mixed. Some find relations using both measures and ToM (e.g [25]), whereas others report that neither measure relates to ToM (e.g [26]). Still, the absolute frequency of parental MST measure has been the MST measure most reliably associated with children’s ToM [15, 16].

It seems reasonable that parents’ variation in speech, within a given category, will direct children’s attention to different aspects of that category of mental state, thereby enriching the spectrum of mental state conditions. However, it seems as if parental MST vocabulary size is a less-reported measure. We have only found one study analyzing the number of different MST words, i.e., the size of MST vocabulary a parent uses while interacting with their child [27]. In their study, absolute emotion word frequency and the number of different emotion words (or vocabulary size) were related to emotion understanding. However, no measure of cognition or desire words used or their respective vocabulary sizes were included in the study. Therefore, we see vocabulary size as a potentially relevant parental MST measure that has been unexplored concerning ToM development.

The three measures of parental MST use (i.e., absolute frequency, proportion, and vocabulary size) highlight different aspects of parent-child interaction. For instance, relatively high absolute frequency of MST use might be more appropriate at a certain age, whilst relatively high proportional parental use of MST words might be more appropriate at a later age. Similarly, the parental MST vocabulary size might be associated with ToM development only at certain ages. A parallel investigation into the associations between parental MST and childrens ToM might give new insights to factors related to ToM development.

Other social factors have also been related to the development of ToM, and it is wise to control for them when investigating associations between ToM and MST. More specifically, socioeconomic status (SES) and the number of siblings positively relate to FB [15] and more general social understanding [28, 29]. For instance, on average, high parental SES has been associated with slightly better child FB [15]. Furthermore, ToM’s association with the number of siblings is thought to result from siblings making the child encounter other perspectives more frequently than those without siblings [15]. However, relations between SES, siblings, and ToM have attenuated over the years, with early publications reporting stronger relations than later publications [15].

Social factors aside, individual factors, such as language ability and executive function (EF), are also important to control when studying associations between MST and ToM. Language and ToM develop together, and Milligan et al.’s [30] meta-analysis found that the relationship is present across many types of language measures, vocabulary perhaps being the “purest” (p. 636). The strength of the relationship was nevertheless highly variable across studies. However, Milligan et al. [30] emphasized the glaring lack of longitudinal studies investigating language in relation to ToM. Therefore, in the present longitudinal study, we attempted to relate vocabulary (measured by the MacArthur Communicative Development Inventories) to ToM.

Besides various language measures, EF development is also related to ToM in preschool ages [31]. The Dimensional Change Card Sort task (DCCS) [32–34] has previously been used to investigate EF in relation to ToM measures. It measures cognitive flexibility [33] which is related to inhibition and working memory [34, 35]. Carlson and Moses [36] found a positive relationship between DCCS and ToM performance, later confirmed by Devine and Hughes [31]. Thus, we will include a measure of DCCS as it appears relevant in investigating associations between EF and ToM.

## Aim and hypotheses

Previous research has reported occasional associations between parental use of emotion and desire MST words and children’s ToM. However, the most reliable relations have been reported between parental use of cognitive words and children’s ToM. Additionally, the most consistent finding is that absolute frequency of parental use of MST words are more reliably related to ToM development compared to proportional parental use of MST words [15, 16]. Nonetheless, parental MST vocabulary size, which we believe to be a likely factor in ToM development, has never been included in an investigation between parental MST and children’s ToM. Therefore, we aimed to investigate absolute frequency, proportions, and vocabulary size of parental use of MST words, whilst separating the measure of MST into cognition, emotion, and desire.

Furthermore, we aimed to control for individual differences in language, SES, sibship size, and EF when investigating associations between parental use of MST words and children’s ToM. In particular, this is the first time longitudinal relations between repeated measurements of MST and ToM are investigated using the ToM scale [8].

Firstly, we hypothesize that associations between children’s ToM and parental MST will be readily found with cognition words, and less so with emotion and desire words. Secondly, we hypothesize children’s ToM will be more readily associated with absolute measures of parental MST use, compared to proportional parental use of MST words. Lastly, we hypothesize that parental MST vocabulary size will be associated with children’s ToM.

## Method

### Recruitment, and attrition

All children were recruited via the Swedish registry, “Statens personadressregister” (SPAR), which includes all persons registered as residents in Sweden. We wanted to include families living in, or around the city of Gothenburg (West Sweden) with children born in October, November, or December of 2014 or January or February 2015. Zip codes were used, striving for a variation in urban/rural high/low socioeconomic status. The original aim was to include about 200 children in the final sample to account for the risk of large attrition between measurements. We expected around a 10% response rate to the invitation letter. Therefore, to ensure we got enough responses, we asked for 3000 addresses and got 2920 unique addresses (because 80 of the addresses we received were duplicates). A total of 230 families replied and gave informed consent. The collection was planned to start when the oldest participant turned 2 years of age. However, the first assessment was delayed and could not start until the children were around two years and four months. This four-month lag was also kept at all follow-up assessments. We still refer to the children at the data collection times as 2-, 3-, 4- and 5-year-olds. We allowed participants to participate all days and times of the week from late December to late July, and we managed to test 180 children when the children were two years of age. At 3 years of age, 150 families participated; at 4 years of age, 136 families participated; at 5 years of age, because of the Covid-19 pandemic in April 2020, 54 families participated.

### Exclusion criteria

After disregarding datapoints recorded from the 44 participants that did not return for testing at 4 years of age, a number of exclusion criteria were implemented. We excluded children not having Swedish as their first language (*n* = 15), inaudible speech, or parents speaking another language than Swedish during MST (*n* = 8) and children with hearing or vision impairments (*n* = 2). Finally, we excluded families that did not have the same parent present at all measurements to ease the interpretation of the results (*n* = 30). One additional child was excluded since it did the opposite of what was instructed when tested at 3 years of age. Thereby, a total of 56 participants were excluded. See Table 1 for a summary of the sample demographics.

### Participants

After attrition and applying the exclusion criteria mentioned above, we included 80 participants (52 girls) at each measurement year; however, testing at 5 years of age was halted before completion because of the Covid-19 pandemic in April 2020. Therefore, only 32 participants (20 girls) were tested at 5 years of age. The included participants’ mean age in months at each year of testing was 28.0 (SD = 0.8, Range = 26.2–30.3) at 2 years of age, 40.4 (SD = 1.0, Range = 37.7–43.7) at 3 years of age, 52.3 (SD = 0.9, Range = 51.1–54.7) at 4 years of age, and 64.1 (SD = 0.5, Range = 62.9 − 65.0) at 5 years of age.

**Table 1:** Sample demographics

	All tested participants at 2 y.	Participants tested at 4 y.	Participants excluded at 4 y.^a^	Participants included in the current study
N	180	136	56	80
% of the baseline sample	100%	76%	31,1%	44,4%
Mean age in years (SD)	2.33 (0.07)	4.36 (0.07)	4.35 (0.07)	–
% girls	56.1%	58.8%	50,0%	65,0%
% with older siblings	64.4%	61.8%	60,7%	62,5%
% mothers with BD +	71.7%	77.9%	80,4%	76,3%
% partners with BD +	50.5%	55.1%	62,5%	50,0%
% parents with an avg. of a BD +	48.9%	53.7%	57,1%	51,3%
% multilingual homes	30.0%	28.7%	46,4%	16,3%
% Swedish as first language	87.7%	89.0%	73,2%	100%

*y. *years old, *BD + *Bachelor’s degree or higher

^a^Excluded based on criteria specified in the Method section

### Materials

This manuscript contains ToM scale, language, SES, and EF data being prepared for submission elsewhere (Sehlstedt & Hjelmquist: Theory of mind development in Swedish preschoolers: A longitudinal investigation. Unpublished; Sehlstedt & Hjelmquist: Developing Theory of Mind in Relation to Executive Function, Socioeconomic Status, Language and Temperament. Unpublished). Additionally, each measurement year included additional tests not presented in this manuscript. Importantly, the perspective in the current manuscript relates the ToM scale to social factors such as the number of siblings and MST, has not been prepared or presented elsewhere.

#### Demographic questionnaire

The parental SES level was measured as the mean of parental educational attainment ranked on a 7-point scale utilizing the Hollingshead index [37]. The point scale was divided into (1) Less than 9 years of primary education, (2) 9 years of primary education, (3) high school (or Gymnasium in Sweden), (4) post-high school education (or Advanced Higher Vocational Education, Higher Vocational Education or Folk High School in Sweden), (5) Bachelor’s degree, (6) Master’s degree, and (7) graduate professional training.

#### Parental Mental State Talk (MST)

In a video-recorded and audiotaped session, the parent was presented with a plastic binder encompassing 10 pictures with more or less emotionally and socially charged situations, such as a child making an angry face towards a peer or two children smiling at a cameraman (pictures from [17]). The parent was asked to talk about what was happening in the pictures, and to switch to the next picture as soon as the child showed that it wanted to turn the page. The dialogue was later transcribed and coded by the authors of this paper, one more experienced researcher, and seven trained students, using a detailed transcription manual. The transcriptions were verbatim, adding minor details to ease the computerized MST extraction.

The MST extraction was conducted on the transcribed dialogues. First, the transcribed material was analyzed using Matlab (R2017a) and in-house written code. In pure computational terms, the Matlab code read the original transcripts (individually) and partitioned the text into individual words. Each word was then compared to all other words to compute how many times and how many different words had been spoken. A total list of words spoken, across all subjects, was then examined manually to make sure that all irrelevant non-word utterances (e.g., “mmm”, “ahaa”, “ooh”) had been excluded. Next, using Ensor and Hughes’ [21] approach, mental state categories including all references to cognitive terms (e.g., “think” or “know”), emotions (e.g., “happy”, “sad”, or “surprised”), and desires (e.g., “want”, “like”, or “hope”) were counted. A complete list of the MST words that were said by the parents and counted is presented in Supplementary Table 1, Additional file 1. Proportions of MST words uttered by parents in each category were calculated as the absolute frequency of MST words in each category divided by the total number of words spoken. Finally, the vocabulary size for each category of MST words spoken by the parent was calculated as the number of different MST words spoken by parents. More specifically, if a parent used the emotion words “happy”, “happier”, and “happiest”, then this would be counted as three different words spoken by the parent. Noteworthy, we had only 20 instances (out of 1280 measured) where a parent used more than 1 inflection of a word, and no parent ever used more than 2.

MST was measured at 2- and 3 years age. The talking time measured in minutes was comparable across measurements in terms of mean duration and standard deviation: at 2 years of age (M = 8.7, SD = 3.3, Range = 2.1–22.3), and at 3 years of age (M = 8.2, SD = 2.4, Range = 3.4–14.0).

#### ToM ability

The ToM scale we used consisted of four steps (Diverse Desire, Diverse Belief, Knowledge Access, Contents False Belief). The test was administered in accordance with the standard procedure by the first author [8]. Each step is presented using a short story together with props (e.g., pictures, dolls, and boxes). The stories for each step can be summarized as follows:

1. Diverse Desires (DD) – The participants are supposed to understand that others may not have the same preferences as themselves regarding food.
2. Diverse Belief (DB) – The participants are supposed to realize that others may not have the same beliefs as themselves regarding where a cat can be hiding.
3. Knowledge Acquisition (KA) – The participant learns something odd about the contents of a box and should recognize that others might not know the contents of that box.
4. Content False Belief (CFB) – The participant should understand that things are not always as they seem and that even if the participant knows what is true, others might not.

The participant was asked to answer a test question, and for Knowledge Access and Contents False Belief, also a control question. Both the control question, when applicable, and the test question must be answered correctly for the child to score 1 for the step. If the child fails either one, the score will be 0. Therefore, the highest possible total score was 4. Testing took approximately 12 min.

#### EF test

DCCS [33] was used to measure EF. During this task, the child is asked to sort cards having two dimensions: shapes and colors. The cards usually have one out of two shapes (e.g., a rabbit or a boat), and these shapes have different colors (e.g., blue or red). There are two versions of the cards that are sorted (e.g., one version that has a red rabbit and the other with a blue boat) and two versions of the cards that are attached to two individual sorting trays (e.g., a blue rabbit, and the other with a red boat). During each stage, children were asked to sort the cards based on rules conveyed by the experimenter (i.e., sort by color or shape). If the child sorted more than 4 cards correctly in the first phase (the pre-switch phase) and sorted all six cards, then the child will proceed to the post-switch phase. If the child sorted five cards (or more) correctly in the post-switch phase, and sorted all six cards, they completed the test. Each completed stage gave an increased score of 1. That means that a successfully completed pre-switch only, or a post-switch phase as well, scored 1, or 2, respectively. This task was used at 2 years of age and took approximately 8 min to complete.

#### Language measurement

A Swedish version of the MacArthur Communicative Development Inventories [38–40] was used to assess the children’s communicative skills. These Swedish Early Communicative Development Inventories (SECDI) are based on parental reports. We used the second version of the SECDI (appropriate for children between 16 and 28 months), which included productive vocabulary. We made a short version (431 words in total) encompassing 13 categories of the complete questionnaire, namely, sound effects and animal sounds, toys, playtime and routines, places to go to, food and beverages, pronouns, words about time, numbers and objects, humans, prepositions and places, verbs, conjunctions and questions, and actions. In addition, the Swedish word “tror” (believe) was added to the existing words included in the form. The form was scored on the total number of different words produced by the child (i.e., the vocabulary as rated by the parent). This form was used when children were 2 years of age. The questionnaire was answered at home and took approximately 35 min to complete.

### The longitudinal design

This study includes data from when the children were 2, 3, 4, and 5 years of age. Measurements at 2 years of age included EF, language, SES, and number of siblings. Measurements at 2 and 3 years of age included MST. Finally, we included measures of ToM at 3, 4, and 5 years of age. Descriptives for all measures are presented in Table 2, and sample sizes for each measure and year are found in Supplementary Table 2, Additional file 1.

**Table 2:** Mean, SD, range, of included variables

Measure	2 years of age		3 years of age		4 years of age		5 years of age	
	Mean (SD)	Range	Mean (SD)	Range	Mean (SD)	Range	Mean (SD)	Range
Parental MST measures								
Abs. Cog.	12.28 (8.67)	0.00–36.00	18.49 (12.41)	0.00–49.00	–	–	–	–
Abs. Emo.	4.05 (3.95)	0.00–16.00	5.26 (3.62)	0.00–14.00	–	–	–	–
Abs. Des.	3.78 (3.28)	0.00–12.00	3.06 (2.59)	0.00–11.00	–	–	–	–
Prop. Cog.	1.94 (1.23)	0.00–5.46	2.88 (1.50)	0.00–7.05	–	–	–	–
Prop. Emo.	0.69 (0.60)	0.00–2.12	0.92 (0.62)	0.00–2.34	–	–	–	–
Prop. Des.	0.58 (0.45)	0.00–1.71	0.52 (0.42)	0.00–1.63	–	–	–	–
Voc. Cog.	3.03 (1.54)	0.00–7.00	3.44 (1.47)	0.00–7.00	–	–	–	–
Voc. Emo.	2.09 (1.53)	0.00–7.00	2.76 (1.66)	0.00–7.00	–	–	–	–
Voc. Des.	1.19 (0.66)	0.00–3.00	1.35 (0.94)	0.00–4.00	–	–	–	–
Child measures								
ToM	–	–	1.98 (0.62)	0.00–3.00	2.78 (0.78)	1.00–4.00	3.36 (0.70)	2.00–4.00
EF	0.68 (0.47)	0.00–1.00	–	–	–	–	–	–
Language^a^	2.15 (0.88)	0.12–3.89	–	–	–	–	–	–
Social factors								
SES	5.54 (0.94)	3.00–7.00	–	–	–	–	–	–
Nr. Siblings	0.71 (0.64)	0.00–3.00	–	–	–	–	–	–

*Abs.* Absolute frequency, *Prop.* Proportion (i.e., %), *Voc.* vocabulary size, *Cog.* Cogitive words, *Emo. *Emotion words, *Des. *Desire words;

^a^Variable is divided by 100

### Procedure

Testing was conducted during December 2016 to April 2020. All pariticipants were accompanied by at least one parent. The first author tested all participants at the Infant and Child Laboratory (INCH) at the Department of Psychology, University of Gothenburg, Gothenburg, Sweden.

The parent started by filling in the short demographic questionnaire. After that, their conversational MST was recorded. The parent and child were left alone in a room while the experimenter waited outside. Once the conversation ended, the experimenter returned to the room and tested EF, and ToM, in that order. The parent then received the SECDI form and was asked to complete the questionnaire at home and send it back by mail. Participants were compensated for their trip to and from the university using standard rates, but no other compensation was offered.

### Data preparations

Potential outliers were identified using boxplots. All variables measured on a scale with less than 7 possible values were not considered eligible for outlier removal. Therefore, the SES, the productive language, and all the MST variables were investigated for outliers. The 27 outliers were spread across 19 participants (6 participants with 2–3 outliers) and 14 variables, namely: SES (1 outlier); absolute cognition at age two (2); absolute emotion at ages two (5), and three (4); absolute desire at age three (3); the proportion of cognition at age two (1); the proportion of emotion at ages two (3), and three (2); the proportion of desire at ages two (1), and three (3); cognition vocabulary size at ages two (1); emotion vocabulary size at age three (1). Means, standard deviations, and ranges for data without outliers are presented in Table 2. Means, standard deviations, range after outlier removal, and skewness and kurtosis values before and after outlier removal per variable are presented in Supplementary Table 2, Additional file 1. Supplementary Figs. 1 and 2, Additional file 1, show the data before and after outlier removal. We report analyses on data without outliers.

Missing values were handled using a full information maximum likelihood approach where all cases are kept in the data (instead of removing incomplete cases) when computing the covariate matrix used for approximating missing values (i.e., FIML.x). The productive language measure was divided by 100 for the structural equation modelling analyses to make variances more equal.

Regarding data preparation, multivariate and univariate normality was assessed for all variables using the Henze-Zirkler (or HZ), and Royston test of multivariate normality. Both tests rejected some univariate and all multivariate normality. The problematic variables were measures of ToM, EF, the number of siblings, and vocabulary MST measures.

### Statistical analyses

**Fig. 1:**
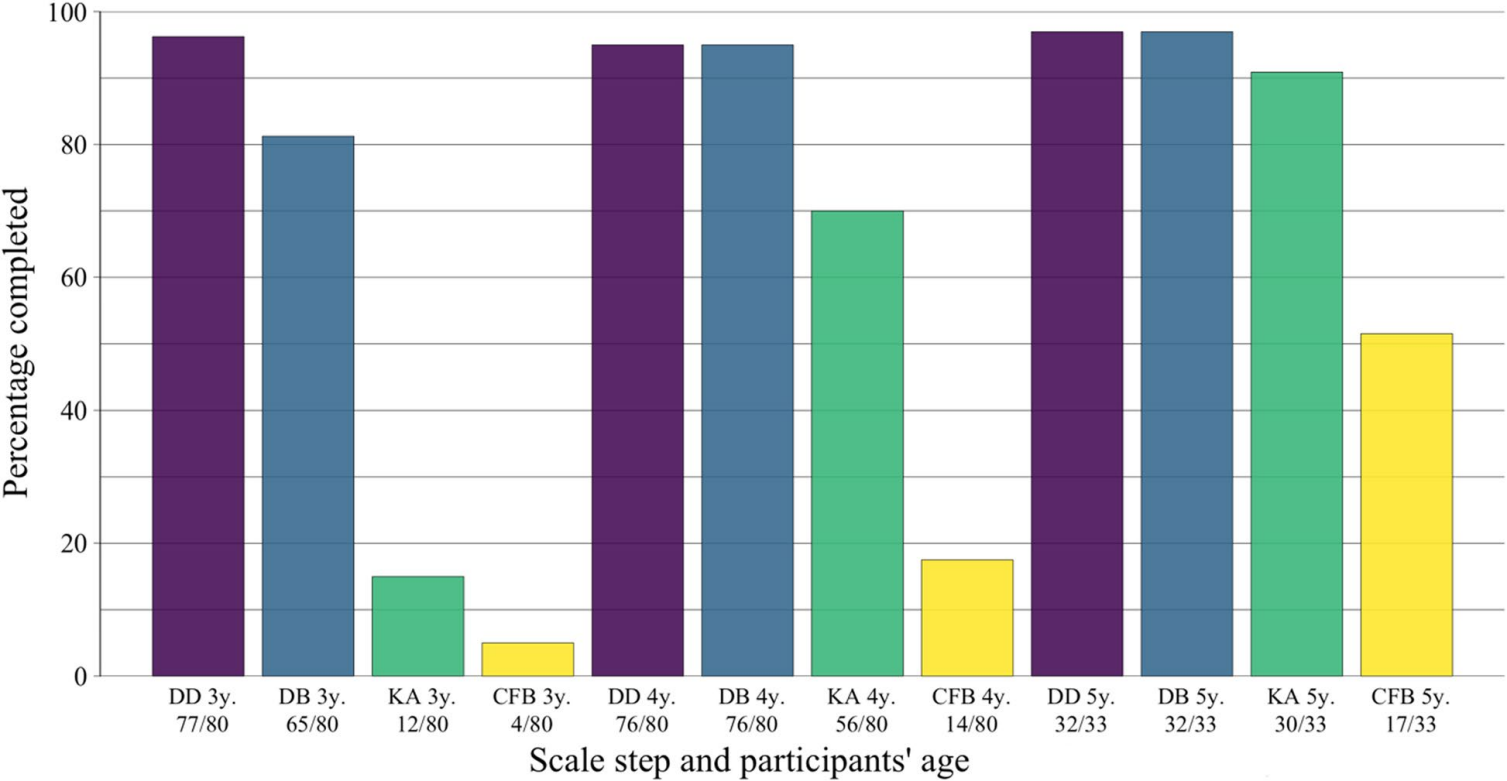
Percentage of Successfully Completed ToM Scale Steps For Each Year Measured. *Note.* DD = Diverse Desires; DB = Diverse Belief; KA = Knowledge Access; CFB = Content False Belief. y = years of age

**Fig. 2:**
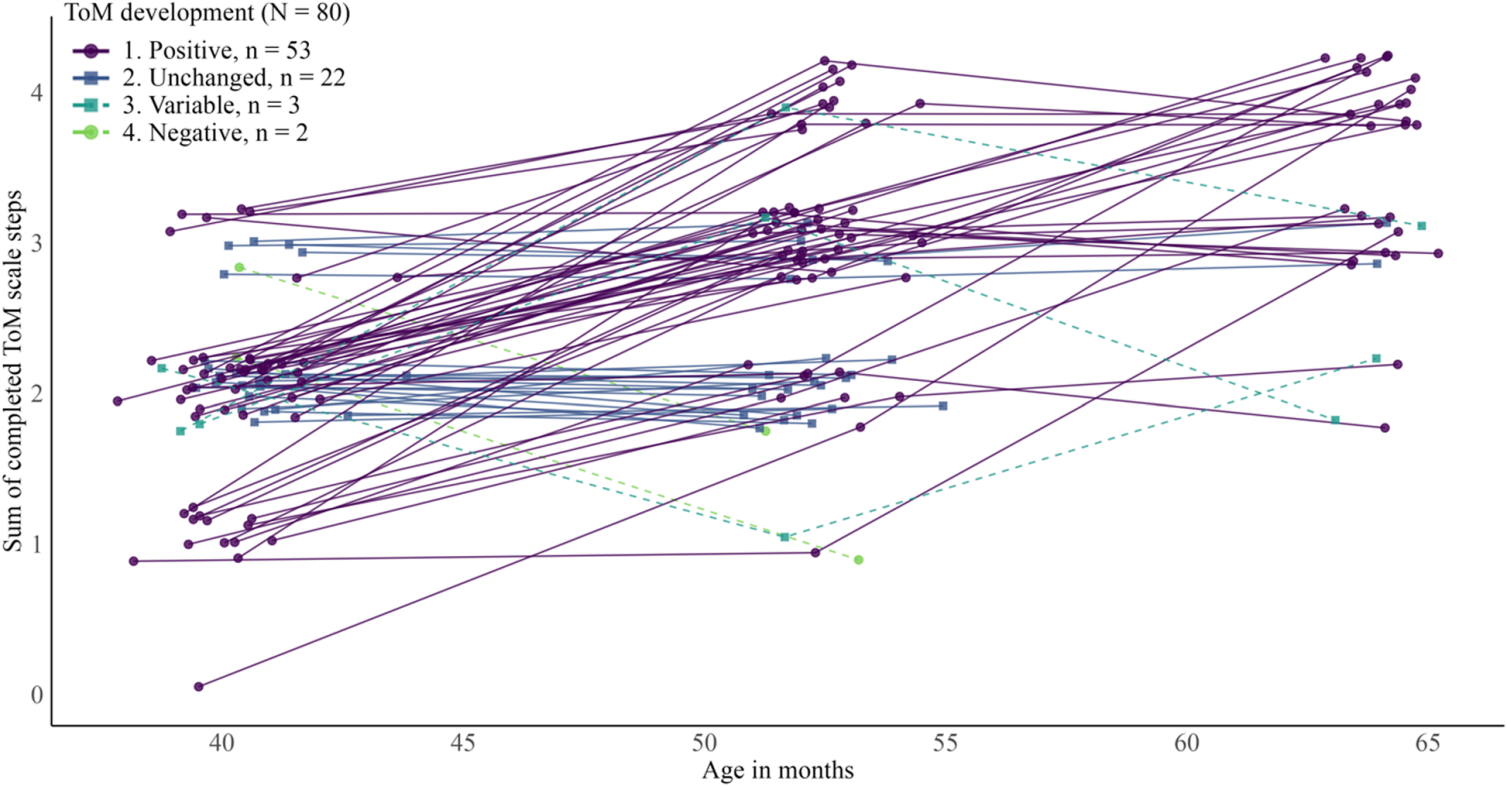
Longitudinal Trajectories of ToM, Separated by Their Developmental Patterns. *Note.* Participants are colored based on their developmental trajectory between their three measurement points. Participants were grouped based on change scores between the measurement at 3 and 4 years of age and 4 and 5 years of age, respectively. A positive development was participants that had a positive change score between at least two measurements and no negative change scores. Negative development was participants that had at least one negative change score and no positive change scores. Unchanged development had no positive and no negative change scores. Only 33 participants could get a variable change score since only 33 were measured at 5 years of age. Variable development had one positive and one negative change score. Participants measured at 3 and 4 years of age and not 5 years of age could only be grouped into positive, negative, or unchanged, as only one change score could be calculated. ToM scores are jittered with a value between − 0.2 and + 0.2 to make data points better visible (i.e., the only possible scores on the scale are 0, 1, 2, 3, and 4). Age in months is displayed as measured and is therefore not jittered

All analyses were defined as significant below an alpha level of 0.05. First, zero-order correlations were calculated to assess all associations between ToM and control variables, with MST (Table 3). Second, Guttman scalogram analyses were performed [41–44] to test the ToM scale’s scalability and reliability across the whole sample [8]. The metrics used to interpret the scale’s scalability are Reproducibility and Index of consistency. Reproducibility is a more lenient measure of a scale’s reproducibility, and the Index of consistency is a more stringent indicator of the scale’s consistency. Acceptable values for the metrics are Reproducibility > 0.9 and Index of consistency > 0.5. Third, basic analyses of the percentage completed steps at each year of measurement were inspected (Fig. 1), and longitudinal trajectories were depicted to inspect development in various aspects of the ToM scale (Fig. 2).

**Table 3:** All spearman correlations excluding correlations between MST variables

Measure	ToM 3y.	ToM 4y.	ToM 5y.	EF	Lang	SES	Sib.
ToM 3y.							
ToM 4y.	0.308*						
ToM 5y.	0.332	0.402*					
EF	0.184	0.156	0.122				
Lang	0.124	0.285*	0.288	0.184			
SES	0.301*	0.121	0.336	− 0.083	0.040		
Nr.Sib	0.022	− 0.102	− 0.032	− 0.037	− 0.193	0.076	
Absolute frequency							
Cog. 2y.	− 0.235*	− 0.067	0.206	− 0.177	0.103	− 0.088	− 0.122
Emo. 2y.	0.019	− 0.017	0.234	0.109	− 0.003	− 0.029	− 0.020
Des. 2y.	− 0.023	− 0.036	0.235	− 0.094	0.046	− 0.140	− 0.059
Cog. 3y.	− 0.094	0.213	0.145	− 0.052	0.105	0.039	− 0.096
Emo. 3y.	− 0.021	0.054	0.271	− 0.028	0.099	0.192	− 0.018
Des. 3y.	0.026	0.089	0.095	− 0.086	0.227	0.082	0.104
Proportion							
Cog. 2y.	− 0.229*	− 0.038	0.087	− 0.151	0.027	− 0.007	− 0.191
Emo. 2y.	0.037	− 0.049	0.306	0.143	0.062	0.068	0.095
Des. 2y.	0.034	0.004	0.150	− 0.001	0.086	− 0.054	− 0.103
Cog. 3y.	− 0.018	0.263*	0.064	− 0.013	− 0.010	− 0.016	− 0.013
Emo. 3y.	0.014	0.124	0.360	0.106	0.015	0.154	0.028
Des. 3y.	− 0.015	0.019	− 0.130	− 0.055	0.179	0.069	0.144
Vocabulary size							
Cog. 2y.	− 0.078	− 0.171	− 0.062	− 0.178	− 0.121	− 0.092	− 0.143
Emo. 2y.	− 0.094	0.026	0.342	0.073	0.091	0.015	0.057
Des. 2y.	0.000	− 0.162	0.155	0.021	0.039	0.015	0.076
Cog. 3y.	0.043	0.314**	0.084	− 0.113	0.089	0.068	− 0.172
Emo. 3y.	0.089	0.175	0.557***	0.046	0.201	0.191	− 0.185
Des. 3y.	− 0.009	0.209	0.149	− 0.071	0.063	− 0.017	− 0.036

*ToM* Theory of mind, *y. *Years of age, *Lang. *Productive language, *SES *Socioeconomic status, *Sib. *Number of older siblings, *EF *Executive function, *Des. *Desire, *Emo. *Emotion, *Cog. *Cognition

* *p* < .05; ** = *p* < .01; *** = *p* < .001

#### Longitudinal latent analyses

As the data was found to be non-normally distributed, robust estimators were used in the analyses. The structural equation modelling analyses were computed using the robust (Hubert-White) maximum likelihood estimator (MLR). MLR has been found to perform well in small sample sizes (< 200) with non-normal distributions and missing data [45, 46] in contrast to alternatives in larger sample sizes [47]. We chose to use MLR for that reason. All robust estimators are marked by a raised letter r (i.e., ^r^).

The models were evaluated using goodness-of-fit (GOF) measures. We used GOF measures that are generally recommended (e.g [48]. The GOF measures and cut-offs we used were a Comparative fit index (CFI) > 0.9, Tucker Lewis Index (TLI) > 0.9, standardized root mean square residual (SRMR) < 0.09, a Root mean squared error of approximation (RMSEA) < 0.05, and, given our small sample size, a not significant Chi^2^ [49–51].

Longitudinal statistical analyses, such as latent growth curve models (LGCM), can analyze individual differences in the level of ability and rate of individual change in that ability when measured more than 2 times [52]. As we measured ToM at 3, 4, and 5 years of age, we used LGCM to analyze and model the level (i.e., individual ToM ability at 4 years of age) and change (i.e., individual rate of change in ToM ability from 3 to 5 years of age) of ToM development. Additionally, LGCM makes it possible to analyze if other variables can predict the level or change. In our case, we are interested to see if MST can predict the level or change in ToM.

Guttman scalogram analyses were performed in Excel. All other analyses were performed in R (R version 4.0.3; [53]), using Rstudio (ver 1.3.1093; [54]). Basic multivariate normality tests were performed using the MVN package [55]. LGCMs were applied and evaluated using the packages lavaan [56], semPlot [57], and semTools [58]. In addition, the first author tested all models, and all authors were involved in stages of the model specification.

## Results

### ToM scale analyses

Basic analyses of the percentage of successful completion per scale step and year (Fig. 1) show ceiling effects for DD at all years and DB at 4 and 5 years of age. KA performance increases most from 3 to 4 years of age, and CFB performance has the largest percentage increase between 4 and 5 years of age. Longitudinal trajectories were stable and most often positive (Fig. 2).

Guttman scalogram analyses revealed that the current data had a Reproducibility of 0.978 and an Index of Consistency of 0.509. These results suggests that the ToM scale is appropriate and highly scalable as a 4-step scale for our sample.

### MST descriptives

**Fig. 3:**
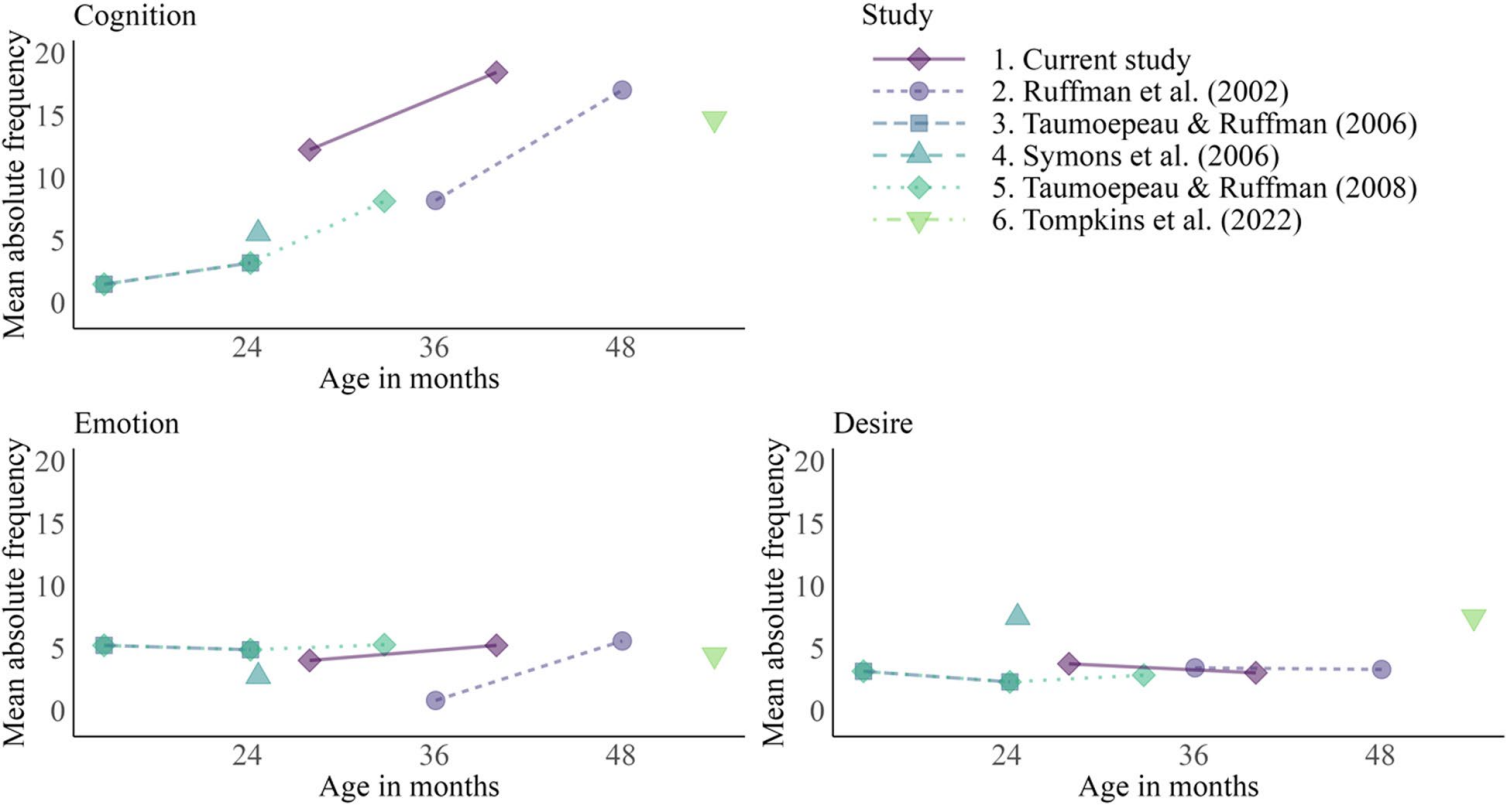
Descriptive comparison Between Previous MST Findings and the Current Results. Note. The Figure includes comparison studies that presented results for all three MST categories (i.e., Cognition, Emotion, and Desire) or equivalent categories as Think/Know instead of Cognition [17] or Affect instead of Emotion [22]. In addition, two comparison studies [22, 59] measured MST at only one timepoint positive relation between proportions of cognition at 3 years of age and ToM at 4 years of age (*p* = .018). Vocabulary size correlations with ToM revealed that cognition vocabulary at 3 years of age was positively related to ToM the year after (*p* = .004) and Emotion vocabulary at 3 years of age was related to ToM at 5 years of age (*p* < .001)

Some descriptive findings are worth mentioning when comparing MST findings in previous research with the current study (Fig. 3). The current study found that parents talk much more about cognition words in comparison to previous studies. However, the increase in cognition words spoken by parents is similar to previous studies. Conversely, our emotion and desire results fit well with previous findings. Additionally, emotion and desire mentioned by parents do not seem to increase on average between the ages of 1 and 4.

### Correlations

Significant correlations between MST and ToM were found for proportions and vocabulary size, but not for absolute frequency values (see Table 3). We found a negative relationship between proportion of cognition at 2 years of age and ToM at 3 years of age (*p* = .043), and a positive relation between proportions of cognition at 3 years of age and ToM at 4 years of age (p = .018). Vocabulary size correlations with ToM revealed that cognition vocabulary at 3 years of age was positively related to ToM the year after (p = .004) and Emotion vocabulary at 3 years of age was related to ToM at 5 years of age (p < .001). 

### Latent growth curve model of ToM development

The construction of the LGCM was conducted stepwise. First, we compared models of ToM performance with decreasing amounts of constraints (starting with the intercept-only model). The only models with acceptable fit were models where the measured ToM scores were not constrained, and both intercept (average) and variance (individual variation) in level and change were estimated. Our model, therefore, included estimations of average and individual levels, and average and individual change of ToM development. The model was centered at 4 years of age to aid convergence of the model (as estimations of level and change become orthogonal) and to aid interpretation of the model, as all other measures were collected before 4 years of age.

The LGCM of ToM was found to have some appropriate GOF measures, namely, Chi2, CFI, and SRMR, while the others were outside acceptable levels, Chi^2^(1) = 2.077 ^r^, *p* = .15, CFI^r^ = 0.917, TLI^r^ = 0.750, SRMR = 0.052, RMSEA^r^ = 0.114, RMSEA^r^ 95% CI [0 ,0.339]. Both the intercept of level (Est = 2.69, *p* < .001, 95% CI [2.57, 2.82]) and change (Est = 0.70, *p* < .001, 95% CI [0.58, 0.82]) were significant. This is interpreted as the average level of ToM at 4 years of age, and the average change in ToM is different from zero, suggesting an average increase in ToM from 3 to 5 years of age. Additionally, the variance of the level was significant (Est = 0.181, *p* < .001, 95% CI [0.07, 0.29]), but the change was not (Est = 0.91, *p* = .27, 95% CI [-0.07, 0.25]). This suggests that participants vary in level of ToM ability at 4 years of age but that the rate of change from 3 to 5 years of age does not vary substantially. Additionally, there was no significant covariance between level and change (Est = 0.04, *p* = .786, 95% CI [-0.06, 0.14]), suggesting no relation between level and change.

We then added the time-invariant control measurements of SES, EF, language, and the number of siblings to the model. The model showed appropriate fit when including the control variables (SES, EF, language, number of siblings), Chi^2^(5) = 2.681^r^, *p* = .749, CFI^r^ = 1, TLI^r^ = 1, SRMR = 0.03, RMSEA^r^ = 0, RMSEA^r^ 95% CI [0 ,0.11]. In this model, the intercept of change was no longer significant (Est = 0.74, *p* = .07, 95% CI [-0.05, 1.53]), suggesting that SES, EF, language, and number of siblings are, all together, associated with general changes in ToM from 3 to 5 years of age. All other estimates were largely unaffected (see Supplementary Table 3, Additional file 1).

### Associations between ToM and MST

The LGCM was then extended with the inclusion of MST. The results of the three models (one for each type of MST) are presented below. Specifically, we estimated one model for each type of MST, i.e., absolute frequency, proportions, and vocabulary size. The three mental state categories (cognition words, emotion words, desire words at age 2 and 3) for each type of MST were included as predictors of level (i.e., individual ToM ability at 4 years of age) and change (i.e., rate of change in ToM ability from 3 to 5 years of age) in ToM. Additionally, the models included the time-invariant control measurements of SES, EF, language, and number of siblings. The necessary covariance matrix to calculate all analyses can be found in Supplementary Tables 4–6, Additional file 1.

**Fig. 4:**
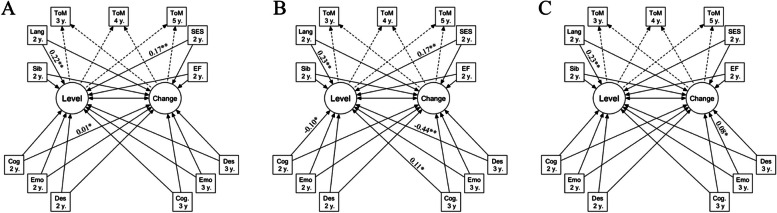
Latent Growth Curve Models Used to Analyze Theory of Mind Development. *Note*. Subfigure A = Absolute frequency of MST; Subfigure B = Proportion of MST; Subfigure C = Vocabulary size of MST; ToM = theory of mind; y. = years of age; Lang = productive language; SES = socioeconomic status; Sib = the number of older siblings; EF = executive function; Des = desire; Emo = emotion; Cog = cognition; Level = variable latent intercept (or individual ToM ability at 4 years of age); Change = variable latent slope (or change in ToM from 3 to 5 years of age). Solid lines indicate regressions, and dashed lines indicate fixed parameters relating to estimating the level and change

Significant associations between ToM and MST are summarized in Fig. 4; Table 4. The complete results for all three models can be found in Supplementary Table 7, Additional file 1. Results of analyses with outliers are summarized in Supplementary Table 8, Additional file 1.

**Table 4:** Summary of significant associations between Theory of Mind (ToM) and MST

		ToM	
		Level (at 4 years of age)	Change (from 3–5 years of age)
MST type	Absolute frequency		Cognition at 2 y. (+)
	Proportion	Cognition words at 2 y. (-) & Cognition words at 3 y. (+) & Desire words at age 3 y. (-)	
	Vocabulary size		Emotion words at 3 y. (+)

*MST* Mental state talk, *Level *Variable latent intercept (or individual ToM ability at 4 years of age), *Change *Variable latent slope (or change in ToM from 3–5 years of age), *y. *Years old, *& *Separating significant findings at the same measurement year, (+) Positive relation, (–) Negative relation

* = *p* < .05; ** = *p* < .01

#### Absolute frequency

The model that included the absolute frequency of parental MST was found to have an appropriate fit across all GOF measures, Chi^2r^ (11) = 9,07, *p* = .615, CFI^r^ = 1, TLI^r^ = 1, SRMR = 0.03, RMSEA^r^ = 0.00, RMSEA^r^ 95% CI = [0.00, 0.10]. In addition, all significant findings associated with the level of ToM were positive, namely the children’s productive language (Est = 0.22, *p* = .005, 95% CI [0.07, 0.37]), and SES (Est = 0.17, *p* = .040, 95% CI [0.01, 0.33]). These results suggest that children with a better than average productive language ability at 2 years of age and children from a family with high SES had higher ToM levels at 4 years of age.

Additionally, parents’ absolute frequency of cognition words was positively related to the change in ToM scores (Est = 0.01, *p* = .035, 95% CI [0.00, 0.03]). This finding suggests that parents who uttered many cognitive words when their children were 2 years of age had children with a slightly faster ToM development than children who did not hear many cognitive words at the same age.

Pseudo R^2^ for level and change was 39% and 44%, respectively.

### Proportions

The model that included proportions of parental MST was found to have an appropriate fit for Chi^2^, SRMR, while CFI and RMSEA were borderline acceptable, and TLI was low, Chi^2r^ (11) = 14.50, *p*^r^ = 0.207, CFI^r^ = 0.89, TLI^r^ = 0.66, SRMR = 0.04, RMSEA^r^ = 0.06, RMSEA^r^ 95% CI = [0.00, 0.11]). The fit for the proportional model is therefore not optimal and the results of this model should be interpred with caution. We found both positive and negative significant associations with the level of ToM. The positive associations included proportions of cognitive words when the child was 3 years of age (Est = 0.11, *p* = .029, 95% CI [0.01, 0.21]), the children’s productive language (Est = 0.23, *p* = .001, 95% CI [0.10, 0.35]), and SES (Est = 0.17, *p* = .024, 95% CI [0.02, 0.31]). These results suggest that parents who mention cognitive words proportionally more often than other parents when the child is 3 years of age had children with higher ToM levels at 4 years of age. These results also suggest (as was found in the absolute frequency analysis) that children with a better than average productive language ability at 2 years of age and children from a family with high SES had higher ToM levels at 4 years of age. The negative associations were both related to MST measurements, namely proportions of cognition words mentioned by parents when the child was 2 years of age (Est = − 0.10, *p* = .033, 95% CI [-0.19, -0.01]), and proportions of parental mentions of desire words when the child was 3 years of age (Est = − 0.44, *p* = .006, 95% CI [-0.75, -0.13]). These results suggest that parents who mention cognitive words proportionally more often than other parents when the child is 2 years of age, and parents who mention desire words proportionally more often than other parents when the child is 3 years of age had children with lower ToM level at 4 years of age.

No significant associations with the children’s rate of change in ToM development were found for parental MST proportions.

Pseudo R^2^ for level and change, was 56% and 74%, respectively.

### Vocabulary size

The model that included the vocabulary size of parental MST was found to have an appropriate fit across all GOF measures (Chi2^r^ (11) = 11.50, *p* = .402, CFI^r^ = 0.98, TLI^r^ = 0.94, SRMR = 0.048, RMSEA^r^ = 0.03, RMSEA^r^ 95% CI = [0, 0.13]).

One significant finding associated with the level of ToM was found, namely a positive association with the child’s productive language (Est = 0.15, *p* = .017, 95% CI [0.03, 0.27]). This result suggests that children with a better than average productive language ability at 2 years of age had higher ToM levels at 4 years of age.

Additionally, the size of the emotion vocabulary used by parents when the child was 2 years of age was positively related to the rate of change in ToM development (Est = 0.08, *p* = .027, 95% CI [0.01, 0.16]). This finding suggests that parents with a more varied emotion vocabulary when their children were 2 years of age had children with a slightly faster ToM development compared to children who did not hear as many different emotion words at the same age.

Pseudo R^2^ for level and change was 53% and 63%, respectively.

## Discussion

We found that parental use of cognitive words was the MST category most often associated with their children’s ToM. Generally, associations were most often found when analyzing proportions of parental MST, followed by vocabulary measures and absolute frequency of parental MST word measures. Nonetheless, only absolute frequency and vocabulary measures of parental MST were associated with the children’s rate of change in ToM development. These findings support and extend previous research [15, 16, 20, 22, 60].

More specifically, regarding links between parental cognitive words and the children’s ToM, the absolute frequency of cognitive words mentioned by parents at 2 years of age was positively related to the children’s rate of change in ToM development. Also, the proportions of spoken cognitive words by parents at 3 years of age were positively related to their child’s ToM level at 4 years. Additionally, the proportions of spoken cognitive words at 2 years of age were negatively related to ToM level at 4 years of age. The results regarding proportions of cognitive words do not fit well with the previous studies summarized by Tompkins et al. [16], as they report that the absolute frequencies of cognitive words spoken to children, and not proportions, were generally found to have a positive association with later FB. Since Tompkins et al.’s [16] summary, others have supported their conclusions regarding cognitive MST [19, 25]. Another study has supported our findings in a cross-sectional design [61]. However, it is important to mention that Carr et al.’s [19] findings are based on the same data as presented by Ruffman et al. [17, 62]. Additionally, previous research on the relation between cognitive words and FB has generally reported significant findings when measuring absolute frequency and for infants, not preschool children [16]. However, it should be noted that our sample of parents used more cognition words compared to previous studies with similarly aged children at both years of measurement. We do not believe this difference stems from SES disparities as we found them comparable to the education-based SES reported by Ruffman et al. [17]. Still, our mean value comparison between the current and previous studies indicated that parents’ use of cognition words increased similarly between 2 and 3 years of age. On a related note, proportions of MST, summed across categories, have been found to have positive relations to later FB and ToM [25, 60]. However, these studies did not report results regarding proportions separating and different types of MST categories.

The negative association between ToM level and proportions of cognitive utterances by parents at 2 years of age that flips to a positive association at 3 years needs an explanation. More specifically, a parent that provides the child with many cognitive words at the early age of 2, but does not give context using non-MST words, might present the child with a less favorable learning situation. An observed example of a parental statement with proportionally high cognitive words whilst context is abstract or lacking in our dataset may be “What do you think they are doing there?”. That type of parental MST talk might result in too high demands on the child’s inference skills. In contrast, an observed example of another parental statement when more context is provided and the statement is less abstract is “Do you think he is tired?”. However, when children have reached the age of 3 their general capacities might be at a level that enables them to benefit from parental input characterized by a large proportion of cognitive words. This line of reasoning aligns conceptually with the suggestion of a parental scaffolding process by Taumoepeau and Ruffman [63], and a recent finding by Tompkins et al. [59], showing that parental MST elicits child MST; however, this is merely our interpretation of the current results and our suggestion is speculative. There is also a possibility that proportions of cognitive words might be a less reliable predictor of ToM, indicated by no relation to the rate of change of ToM. We encourage future studies to investigate the relation between different measures of parental MST and children’s ToM development.

Another negative association was found between the proportion of spoken desire words at 3 years of age and ToM level at 4 years of age. This finding fits well with the suggestion that parental use of desire words is age-appropriate for children younger than 2 years of age [63]. Two recent studies confirmed and expanded this conclusion [25, 64]. However, desire words spoken by parents are generally unrelated to FB in infants or preschool children [16], and there might be an explanation in quality [63, 64] and culture [25] aspects of parental MST. Also, Taumoepeau and Ruffman’s [65] results with children younger than 2 years of age suggest that parental desire language referring to the child’s desires was the “more consistent correlate of mental state language and emotion understanding” (p. 465) in comparison to parents referring to others’ desires. Unfortunately, Tompkins et al.’s [16] meta-analysis could not include enough studies to perform analyses on the effects of referents. Nonetheless, future studies should investigate the relationship between desire talk and ToM.

Measurement of parental MST vocabulary size revealed that vocabulary size in the emotion domain was associated with the children’s rate of change in ToM development. In other words, parents using a larger emotion vocabulary in conversation with their children at 3 years of age had a relatively faster ToM development than children of parents who used a smaller emotion vocabulary. Since this finding is an original finding within ToM research, it is not possible to relate it to previous research in a straightforward manner. However, the meta-analytical findings presented by Tompkins et al. [16] suggest a positive but non-significant relation between emotion MST (in contrast to the significant association with cognitive MST), and ToM. One suggestion is that parents with larger emotion vocabularies might be better at describing the spectrum of relevant emotion states around the child. This suggestion, however, is a topic for future research and replication.

We included some of the variables that have previously been associated with ToM also in our analyses to make them more complete, and found support for SES and child language being associated with ToM. More specifically, we found that the child’s productive language at 2 years of age and SES had a positive relation to ToM level at 4 years. These findings fit well with the meta-analyses that reported positive relations to ToM with SES [15] and language [30]. However, it might be important to note that the current SES and language measures were not associated with the rate of change in ToM development. This stands in contrast to similar analyses in the meta-analysis by Devine and Hughes [15] and can be interpreted as that our SES and productive language measures were unrelated to ToM in analyses that included previous ToM ability as a control, as found in unpublished observations (Sehlstedt & Hjelmquist: Theory of mind development in Swedish preschoolers: A longitudinal investigation. Unpublished) .

Unexpectedly, the number of siblings and EF were unrelated to ToM development regardless of MST type included in the analysis. These findings go against most of the previously published research as (1) the number of siblings has positively related to ToM [15] although there are contradicting results (e.g [66]), and (2) EF are often found to be related to ToM [67, 68]. This discrepancy should be addressed. Firstly, the parents in our study were (on average) highly educated and might therefore differ from most of the samples that have been previously investigated. Secondly, we did not collect the sibling measure with the strongest association to ToM, namely the number of “child-aged” siblings [15], which can be described as siblings on a similar cognitive level, regardless of being somewhat older or younger.

Nonetheless, we used the number of siblings as our measure, and most siblings in our sample were relatively close in age to the participants in our study. However, we have no information on whether they were child-aged siblings. Noteworthy, only one out of the 80 families had more than two siblings, but the mere presence of a sibling might suffice to improve ToM if the interaction is, at least on occasion, positive regardless of the sibling being older or younger [69]. In a more general perspective, in an original study by Downey and Condron [28] and a follow-up by Downey et al. [29] the number of siblings (regardless of proximity in age) was positively related to teacher-rated emotional understanding (e.g., being generally considerate towards others). Still, research suggests that emotional understanding and ToM, or FB, are perhaps best separated (e.g [16, 70]). Therefore, we suggest that there might be unmeasured quality factors in the sibling relationships that might have affected the results originally suggested by Cutting and Dunn [70], and in line with the findings by Hou et al. [69].

Our study is the first to report a longitudinal latent measure of the level and change of ToM development. The analysis is, therefore, no longer a correlation or regression of the actual measured ToM scores but rather unobservable factors meant to capture a purer measurement of the ToM development. Therefore, differences in the analytical methods used in previous research, compared to the current study, might explain the discrepancy in the results. Nonetheless, our findings need to be replicated.

As expected, the ToM scale was reliable as a 4-step scale. This finding is in line with previous studies using the same 4-step ToM scale as in the current study [11, 12, 14] and other variations [13, 71, 72]. The reason why ToM scales with more than 4 steps are not often found to be consistent might be because of the random variance introduced by the study design, as discussed in unpublished observations (Sehlstedt & Hjelmquist: Theory of mind development in Swedish preschoolers: A longitudinal investigation. Unpublished). For example, many cross-sectional studies do not find that the 5- (or more) step scale is consistent, but longitudinal investigations find the scale to be consistent regardless of the number of steps included [73, 74]. It might also be that the scalability of the ToM scale is dependent on the sample measured being of a certain age as the 4-step scale’s consistency seems to be acceptable in the wider, but not more narrow age range, as found in unpublished observations (e.g., only a 2-year span, not including participants 5 years of age; Sehlstedt & Hjelmquist: Theory of mind development in Swedish preschoolers: A longitudinal investigation. Unpublished). Therefore, the combination of a longitudinal investigation including participants older than 4 years of age might be the way to confirm the scale’s reliability and consistency (see [75] for a recent effort).

### Limitations

The current study would have benefitted from a complete set of datapoints on the ToM scale at 5 years of age. However, due to the Covid-19 pandemic, we had to halt testing in March 2020, when more than half of the participants were still scheduled for one last measurement. Additionally, we handled missing values by using a version of maximum likelihood. However, a larger sample size would have increased the quality of the handling of missing values, as maximum likelihood estimations are designed for large datasets [76]. Additionally, even if outliers were removed in our data, we still have minor normality issues in some of our variables, making some results less trustworthy than we aimed for.

Furthermore, measuring grammar, together with a comprehension or a discourse test at 2 years of age, would have made analyses of relations between language and ToM more complete [30, 77]. It would also have made it possible to compare the relative contributions of the different language measures, which is a current topic of discussion [78–83].

Moreover, the measurement of DCCS at 2 years of age had low variance (0–1). Also, all children included in the current study, except for one, were 1–3 months younger than what the original author reports is suitable for the DCCS [33]. This may have resulted in a reduced chance to find associations between our earliest measurement of EF and ToM. However, it might be argued that DCCS at two years of age primarily measures the ability to stick to one dimension, color or shape, and does not tap flexibility or “real EF”, and implies a restricted range of performance. Nevertheless, it is a valid step in the development of EF, reflecting self-regulation, and as such relevant for the issue of emerging ToM skills.

Additionally, the current dataset did not have an acceptable fit on all GOF measures for the model analyzing proportions of MST in relation to ToM. Moreover, given the relatively low sample size, our GOF analyses do not include information to identify ill-fitting models optimally [76]. Therefore, even if our Chi^2^ tests were not significant, the small sample size might have affected our analyses adversely. A related limitation in the current study is that we have limited possibility to find individual differences in the rate of change in ToM development [84]. If possible, future research that will include longitudinal latent analyses when investigating the ToM scale could (1) increase the sample size, (2) measure each step more than once using different scenarios at each testing occasion (e.g., as is now commonly done with FB tests) to capture a more complete and stable measure of ToM, (3) increase the number of (and perhaps vary the time-interval between) measurement points in line with the suggestions by Brandmaier et al. [85], Hertzog et al. [84], and von Oertzen et al. [86].

## Conclusion

We investigated parents’ propensity to talk about others’ cognitive states and found relations to children’s ToM ability in a longitudinal study with repeated measures of MST and ToM. More specifically, parents’ spoken cognitive words are most likely to relate to the child’s ToM level at 4 years of age. This suggests that the parental ability to capture the thoughts, beliefs, and knowledge present in different scenarios is associated with children’s ability to understand other minds. We also report that the child’s ToM development rate from 3 to 5 years of age was associated with the absolute frequency of cognitive words spoken by parents and the emotion vocabulary used by parents at 2 years of age. The trajectory of ToM development is thus related to the verbal input that parents provided years earlier. In sum, the current study’s results expand our understanding of how parents’ way of talking about the mental states of others is associated with their children’s ability to understand and further develop ToM.

### Supplementary Information

**Supplementary Material 1.**

**Supplementary Material 2.**

**Supplementary Material 3.**

**Supplementary Material 4.**

**Supplementary Material 5.**

## Data Availability

The datasets supporting the conclusions of this article are included within the article (and its additional files). See Additional file 2 for the complete dataset with outliers removed and Additional file 3 for the complete dataset with outliers kept in the data. It should be noted that descriptive information that is not included in any analysis (e.g., age, gender, and talking time) has been removed from Additional files 2 and 3. The complete Guttman scalogram analysis is divided into three Supplementary Tables showing the Guttman patterns (Supplementary Table 9, Additional file 4), calculations performed (Supplementary Table 10, Additional file 4) and results (Supplementary Table 11, Additional file 4). See Additional file 5 for the R-script to load Additional file 3, remove outliers, analyze the data, and save the results to files. The spreadsheet used to perform Guttman scale analyses and the code used to extract the different MST types from text is available by contacting the first author.

## Abbreviations

CFB: Content False Belief
CFI: Comparative Fit Index
DB: Diverse Belief
DCCS: Dimensional Change Card Sort task
DD: Diverse Desire
EF: Executive Function
FB: False Belief
FIML: Full Information Maximum Likelihood
GOF: Goodness-of-Fit
HZ: Henze-Zirkler
KA: Knowledge Aquisition
LGCM: Latent Growth Curve Models
MLR: Maximum Likelihood Estimator
MST: Mental State Talk
RMSEA: Root Mean Squared Error of Approximation
SES: Socioeconomic Status
SRMR: Standardized Root Mean Square Residual
TLI: Tucker Lewis Index
ToM: Theory of Mind
