# Supplementary material for: The longitudinal relations between mental state talk and theory of mind
Source: BMC Psychol. 2024 Apr 6;12:191. doi: 10.1186/s40359-024-01692-y (PMC10998333; doi:10.1186/s40359-024-01692-y)
Supplement: Supplementary file 1 — Supplementary Material 1. [file 40359_2024_1692_MOESM1_ESM.docx]

| **Supplementary Table 1** | | | | |
| --- | --- | --- | --- | --- |
| *Description of the MST Vocabulary Used by Parents.* | | | | |
|  | English word | Swedish word | No. inf. | Count |
| **Cognition** | |  |  |  |
|  | Know | Veta | 2 | 1 |
|  | Pretends | Låtsas | 1 | 4 |
|  | Think (believe) | Tro | 2 | 29 |
|  | Think (contemplate) | Tänka | 3 | 5 |
|  | Think (opinion) | Tycka | 3 | 13 |
|  | Think (ponder) | Funderar | 1 | 1 |
|  | Think (wonder) | Undra | 2 | 2 |
| **Emotion** | |  |  |  |
|  | Angry | Arg | 3 | 54 |
|  | Happy | Glad | 3 | 28 |
|  | Sad | Ledsen | 3 | 43 |
|  | Scared | Rädd | 1 | 6 |
|  | Surprised | Förvånad | 1 | 7 |
| **Desire** | |  |  |  |
|  | Like | Gilla | 2 | 10 |
|  | Need | Behöver | 1 | 3 |
|  | Want | Vilja | 2 | 5 |
| *Note.* Count = number of parents mentioning the word at either year of MST measurement. No. inf. = Number of inflections recorded across all recorded parents*.* Eight parents never used any of the words above. Fourteen parents used 1 of the words above at either year of MST measurement, 11 used 2, 14 used 3, 14 used 4, 10 used 5, 2 used 6, 2 used 7, 1 used 8, 2 used 9, 1 used 12, and 1 used 13. | | | | |

| **Supplementary Table 2** | |  |  |  |  |  |  |  |  |  |  |
| --- | --- | --- | --- | --- | --- | --- | --- | --- | --- | --- | --- |
| *Mean, SD, Range, Skewness, and Kurtosis for all Included Variables, Before and After Outlier Removal.* | | | | | | | | | | | |
|  | Outliers kept in data | | | | |  |  | Outliers removed | | | |
| Measure | Mean (SD) | Range | n | Mis. | Skew. | Kurt. |  | n | Mis. | Skew. | Kurt. |
| No. Sib. 2y. | 0.55 (0.61) | 0.00 - 2.00 | 80 | 0 | 0.62 | -0.61 |  | 80 | 0 | 0.62 | -0.61 |
| SES 2y. | 5.49 (1.03) | 1.50 - 7.00 | 80 | 0 | -1.08 | 1.51 |  | 79 | 1 | -0.65 | -0.24 |
| EF 2y. | 0.68 (0.47) | 0.00 - 1.00 | 66 | 14 | -0.76 | -1.44 |  | 66 | 14 | -0.76 | -1.44 |
| Lang 2y. ^a^ | 2.15 (0.88) | 0.12 - 3.89 | 76 | 4 | -0.44 | -0.65 |  | 76 | 4 | -0.44 | -0.65 |
| ToM 3y. | 1.98 (0.62) | 0.00 - 3.00 | 80 | 0 | -0.31 | 0.59 |  | 80 | 0 | -0.31 | 0.59 |
| ToM 4y. | 2.78 (0.78) | 1.00 - 4.00 | 80 | 0 | -0.07 | -0.60 |  | 80 | 0 | -0.07 | -0.60 |
| ToM 5y. | 3.36 (0.70) | 2.00 - 4.00 | 33 | 47 | -0.59 | -0.88 |  | 33 | 47 | -0.59 | -0.88 |
| Abs. Cog. 2y. | 13.50 (11.96) | 0.00 - 82.00 | 80 | 0 | 2.59 | 11.44 |  | 78 | 2 | 0.70 | -0.23 |
| Abs. Emo. 2y. | 5.03 (5.39) | 0.00 - 21.00 | 80 | 0 | 1.42 | 1.34 |  | 75 | 5 | 1.19 | 0.99 |
| Abs. Des. 2y. | 3.78 (3.28) | 0.00 - 12.00 | 80 | 0 | 0.86 | -0.18 |  | 80 | 0 | 0.86 | -0.18 |
| Abs. Cog. 3y. | 18.49 (12.41) | 0.00 - 49.00 | 80 | 0 | 0.52 | -0.69 |  | 80 | 0 | 0.52 | -0.69 |
| Abs. Emo. 3y. | 5.84 (4.34) | 0.00 - 18.00 | 80 | 0 | 0.84 | 0.28 |  | 76 | 4 | 0.51 | -0.27 |
| Abs. Des. 3y. | 3.43 (3.13) | 0.00 - 13.00 | 80 | 0 | 1.20 | 1.17 |  | 77 | 3 | 0.84 | 0.24 |
| Prop. Cog. 2y. | 2.01 (1.36) | 0.00 - 7.31 | 80 | 0 | 1.13 | 1.58 |  | 79 | 1 | 0.70 | -0.28 |
| Prop. Emo. 2y. | 0.77 (0.72) | 0.00 - 3.24 | 80 | 0 | 1.10 | 0.89 |  | 77 | 3 | 0.71 | -0.46 |
| Prop. Des. 2y. | 0.60 (0.47) | 0.00 - 2.07 | 80 | 0 | 0.72 | -0.07 |  | 79 | 1 | 0.54 | -0.64 |
| Prop. Cog. 3y. | 2.88 (1.50) | 0.00 - 7.05 | 80 | 0 | 0.28 | -0.51 |  | 80 | 0 | 0.28 | -0.51 |
| Prop. Emo. 3y. | 0.96 (0.67) | 0.00 - 2.54 | 80 | 0 | 0.58 | -0.37 |  | 78 | 2 | 0.50 | -0.43 |
| Prop. Des. 3y. | 0.60 (0.56) | 0.00 - 3.23 | 80 | 0 | 1.74 | 4.82 |  | 77 | 3 | 0.57 | -0.42 |
| Voc. Cog. 2y. | 3.09 (1.62) | 0.00 - 8.00 | 80 | 0 | 0.47 | -0.01 |  | 79 | 1 | 0.27 | -0.50 |
| Voc. Emo. 2y. | 2.09 (1.53) | 0.00 - 7.00 | 80 | 0 | 0.44 | -0.11 |  | 80 | 0 | 0.44 | -0.11 |
| Voc. Des. 2y. | 1.19 (0.66) | 0.00 - 3.00 | 80 | 0 | 0.05 | -0.28 |  | 80 | 0 | 0.05 | -0.28 |
| Voc. Cog. 3y. | 3.44 (1.47) | 0.00 - 7.00 | 80 | 0 | -0.02 | -0.72 |  | 80 | 0 | -0.02 | -0.72 |
| Voc. Emo. 3y. | 2.76 (1.66) | 0.00 - 7.00 | 80 | 0 | 0.18 | -0.50 |  | 80 | 0 | 0.18 | -0.50 |
| Voc. Des. 3y. | 1.35 (0.94) | 0.00 - 4.00 | 80 | 0 | 0.61 | 0.51 |  | 80 | 0 | 0.61 | 0.51 |
| Note. Abs. = Absolute frequency; Prop = Proportions (i.e, %); Voc. = vocabulary size; Cog = Cogitive words; Emo. = Emotional words; Des = Desire words; Lang. = Productive language; SES = Socioeconomic status as measured by averaged parental education; No. Sib. = Number of siblings; EF = Executive function; ^a^ = variable is divided by 100. | | | | | | | | | | | |

| **Supplementary Table 3** | | | | | | | |  |
| --- | --- | --- | --- | --- | --- | --- | --- | --- |
| *Estimates for the Baseline Latent Growth Curve Model Without and with Outliers.* | | | | | | | |  |
|  | Outliers removed | | |  | Outliers kept | | |  |
| Measure | Est [95% CI] | SE | *p* |  | Est [95% CI] | SE | *p* |  |
|  | Regressions with Level in ToM | | | | | | |  |
| Lang. | 0.18 [0.03, 0.32] | 0.07 | 0.014 |  | 0.17 [0.03, 0.32] | 0.07 | 0.016 |  |
| SES | 0.15 [-0.01, 0.31] | 0.08 | 0.069 |  | 0.09 [-0.05, 0.23] | 0.07 | 0.220 |  |
| No. Sib. | -0.02 [-0.21, 0.18] | 0.10 | 0.879 |  | 0.00 [-0.20, 0.20] | 0.10 | 1.000 |  |
| EF | 0.18 [-0.13, 0.48] | 0.15 | 0.254 |  | 0.20 [-0.10, 0.49] | 0.15 | 0.198 |  |
|  | Regressions with Change in ToM | | | | | | |  |
| Lang. | 0.07 [-0.08, 0.22] | 0.07 | 0.349 |  | 0.07 [-0.08, 0.22] | 0.08 | 0.358 |  |
| SES | -0.02 [-0.14, 0.10] | 0.06 | 0.750 |  | -0.06 [-0.14, 0.03] | 0.04 | 0.186 |  |
| No. Sib. | -0.09 [-0.29, 0.11] | 0.10 | 0.392 |  | -0.08 [-0.29, 0.13] | 0.11 | 0.441 |  |
| EF | -0.02 [-0.31, 0.27] | 0.15 | 0.894 |  | 0.00 [-0.29, 0.28] | 0.14 | 0.985 |  |
|  | Latent factors (ToM) | | | | | | |  |
| Level i. | 1.38 [0.48, 2.27] | 0.46 | 0.003 |  | 1.70 [0.89, 2.52] | 0.41 | <0.001 |  |
| Level v. | 0.14 [0.06, 0.23] | 0.04 | <0.001 |  | 0.16 [0.08, 0.23] | 0.04 | <0.001 |  |
| Change i. | 0.74 [-0.05, 1.53] | 0.40 | 0.067 |  | 0.93 [0.31, 1.54] | 0.32 | 0.003 |  |
| Change v. | 0.11 [-0.04, 0.25] | 0.07 | 0.141 |  | 0.11 [-0.04, 0.26] | 0.08 | 0.143 |  |
| Covar. | 0.04 [-0.05, 0.13] | 0.05 | 0.359 |  | 0.05 [-0.03, 0.14] | 0.04 | 0.244 |  |
| *Note.* ToM = Theory of mind; Level = ToM level at 4 years of age; Change = rate of change in ToM level from 3-5 years of age; Lang. = Productive language; SES = Socioeconomic status as measured by averaged parental education; No. Sib. = Number of siblings; EF = Executive function; i. = intercept; v. = variance; Covar. = Covariance between Level and Change. | | | | | | | |  |
|  |  |  |  |  |  |  |  |  |
|  |  |  |  |  |  |  |  |  |
|  |  |  |  |  |  |  |  |  |
|  |  |  |  |  |  |  |  |  |
|  |  |  |  |  |  |  |  |  |

**Supplementary Figure 1**

*Longitudinal Relation Between Measures of MST and ToM Before Removal of Outliers.*


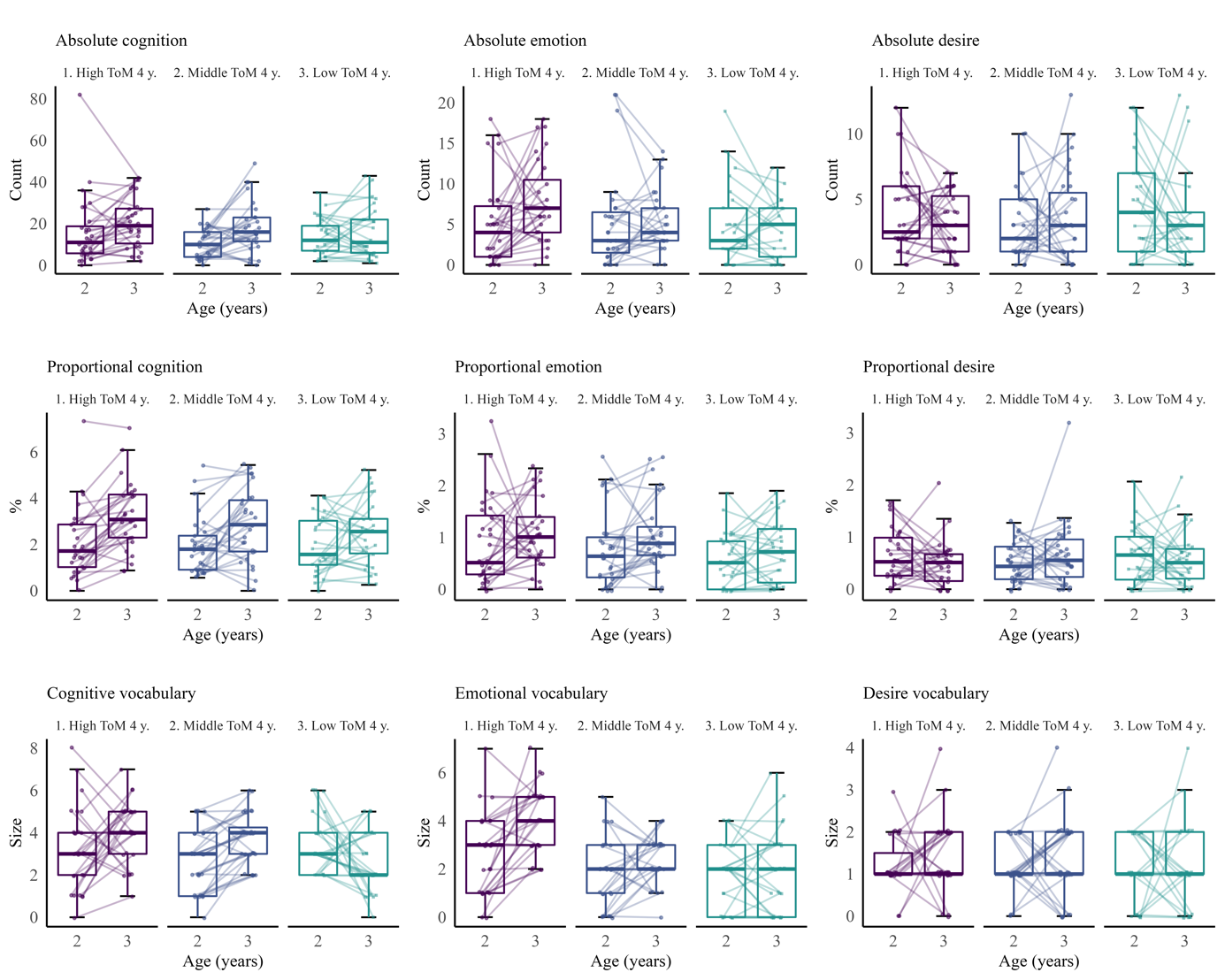


*Note.* For this Figure the sample was divided into high, middle, and low ToM based on their individual latent ToM ability at 4 years of age. The low ToM participants have individual latent level scores below sample mean – (SD/2), high have individual latent level scores above sample mean + (SD/2), and the middle are all participants between low and high. The Y-scales are set individually to aid interpretability between low and high ToM at the expense of ease of comparison between MST categorie

**Supplementary Figure 2**

*Longitudinal Relation Between Measures of MST and ToM After Outliers Were Removed.*


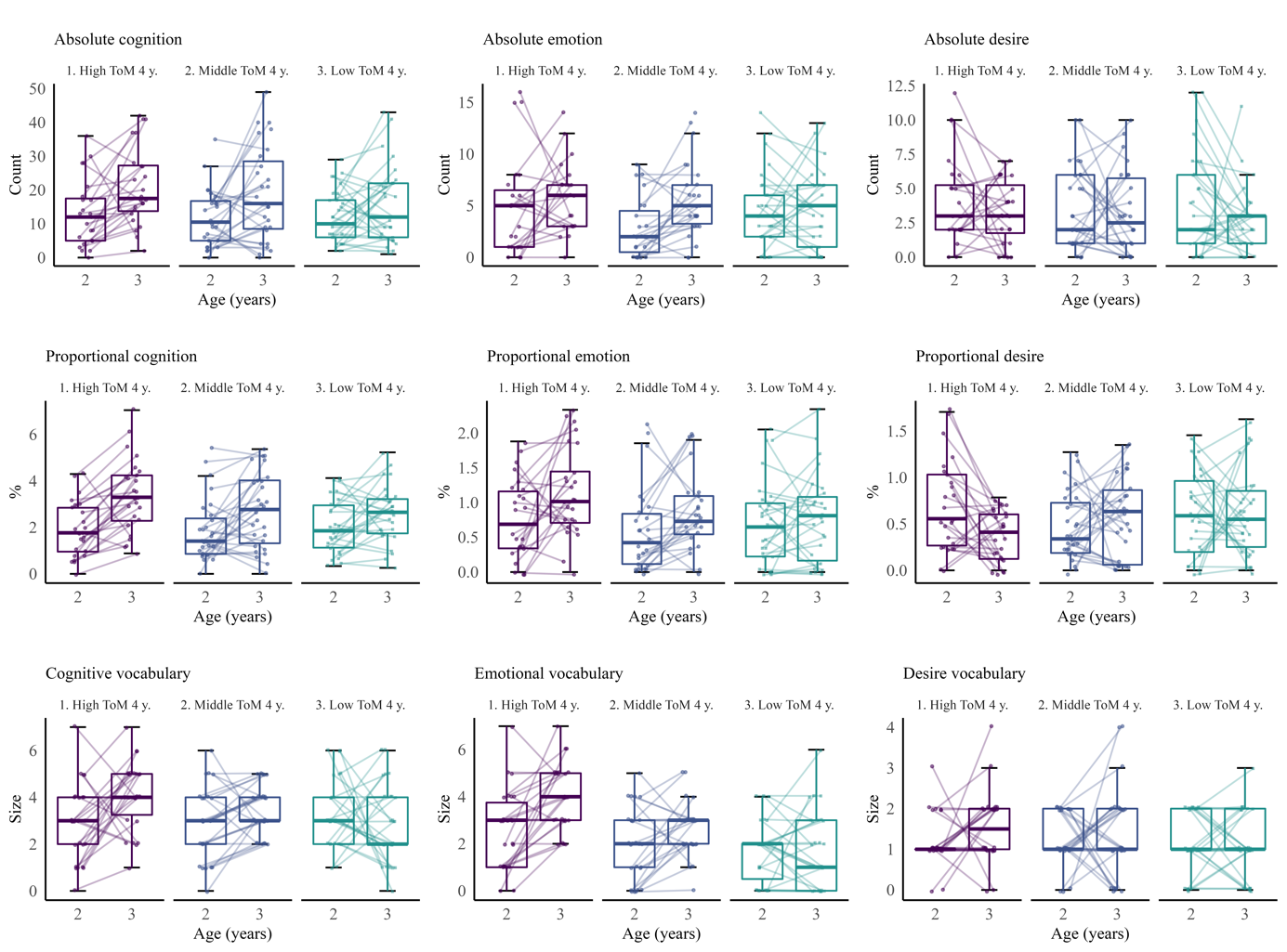


*Note.* For this Figure the sample was divided into high, middle, and low ToM based on their individual latent ToM ability at 4 years of age. The low ToM participants have individual latent level scores below sample mean – (SD/2), high have individual latent level scores above sample mean + (SD/2), and the middle are all participants between low and high. The Y-scales are set individually to aid interpretability between low and high ToM at the expense of ease of comparison between MST categories.

| **Supplementary Table 4** | | | | | | | | | | | | | | |
| --- | --- | --- | --- | --- | --- | --- | --- | --- | --- | --- | --- | --- | --- | --- |
| *Covariance Matrix for the Absolute Frequency of MST Analyses.* | | | | | | | | | | | | | | |
| Variable | 1 | 2 | 3 | 4 | 5 | 6 | 7 | 8 | 9 | 10 | 11 | 12 | 13 |  |
| 1. ToM 3y. | 0.3791 | 0.1462 | 0.1364 | 0.0469 | 0.0811 | 0.1548 | 0.0307 | -1.2654 | 0.0007 | -0.0943 | -0.7978 | -0.0063 | -0.0386 |  |
| 2. ToM 4y. | 0.1462 | 0.6070 | 0.2330 | 0.0552 | 0.1801 | 0.1049 | -0.0402 | -0.0047 | -0.0823 | -0.1652 | 1.5035 | 0.1502 | 0.1452 |  |
| 3. ToM 5y. | 0.1364 | 0.2330 | 0.4886 | 0.0513 | 0.2229 | 0.1542 | 0.0000 | 1.3892 | 0.6744 | 0.3636 | 1.5398 | 0.7724 | -0.0242 |  |
| 4. EF 2y. | 0.0469 | 0.0552 | 0.0513 | 0.2203 | 0.0893 | -0.0364 | -0.0112 | -0.7882 | 0.0653 | -0.2699 | -0.4021 | -0.0275 | -0.1208 |  |
| 5. Lang 2y. | 0.0811 | 0.1801 | 0.2229 | 0.0893 | 0.7770 | -0.0041 | -0.1234 | 0.9142 | 0.2513 | 0.0375 | 1.5485 | 0.2379 | 0.5449 |  |
| 6. SES 2y. | 0.1548 | 0.1049 | 0.1542 | -0.0364 | -0.0041 | 0.8767 | 0.0625 | -0.3391 | 0.1101 | -0.4853 | 0.2710 | 0.6981 | 0.3119 |  |
| 7. No. Sib. 2y. | 0.0307 | -0.0402 | 0.0000 | -0.0112 | -0.1234 | 0.0625 | 0.4100 | -0.6727 | -0.0510 | -0.0275 | -1.0100 | -0.0232 | 0.1372 |  |
| 8. Abs. Cog. 2y. | -1.2654 | -0.0047 | 1.3892 | -0.7882 | 0.9142 | -0.3391 | -0.6727 | 75.1142 | 9.5862 | 11.3623 | 40.1092 | 11.3973 | 2.5721 |  |
| 9. Abs. Emo. 2y. | 0.0007 | -0.0823 | 0.6744 | 0.0653 | 0.2513 | 0.1101 | -0.0510 | 9.5862 | 15.6187 | 2.9775 | 9.9461 | 4.6608 | -1.2949 |  |
| 10. Abs. Des. 2y. | -0.0943 | -0.1652 | 0.3636 | -0.2699 | 0.0375 | -0.4853 | -0.0275 | 11.3623 | 2.9775 | 10.7335 | 8.4782 | 2.9102 | 0.9831 |  |
| 11. Abs. Cog. 3y. | -0.7978 | 1.5035 | 1.5398 | -0.4021 | 1.5485 | 0.2710 | -1.0100 | 40.1092 | 9.9461 | 8.4782 | 154.0252 | 20.3593 | 10.2092 |  |
| 12. Abs. Emo. 3y. | -0.0063 | 0.1502 | 0.7724 | -0.0275 | 0.2379 | 0.6981 | -0.0232 | 11.3973 | 4.6608 | 2.9102 | 20.3593 | 13.1032 | 0.9621 |  |
| 13. Abs. Des. 3y. | -0.0386 | 0.1452 | -0.0242 | -0.1208 | 0.5449 | 0.3119 | 0.1372 | 2.5721 | -1.2949 | 0.9831 | 10.2092 | 0.9621 | 6.6931 |  |

| **Supplementary Table 5** | | | | | | | | | | | | | |
| --- | --- | --- | --- | --- | --- | --- | --- | --- | --- | --- | --- | --- | --- |
| *Covariance Matrix for the Proportions of MST Analyses* | | | | | | | | | | | | | |
| Variable | 1 | 2 | 3 | 4 | 5 | 6 | 7 | 8 | 9 | 10 | 11 | 12 | 13 |
| 1. ToM 3y. | 0.3791 | 0.1462 | 0.1364 | 0.0469 | 0.0811 | 0.1548 | 0.0307 | -0.1933 | 0.0080 | 0.0027 | -0.0198 | 0.0183 | -0.0091 |
| 2. ToM 4y. | 0.1462 | 0.6070 | 0.2330 | 0.0552 | 0.1801 | 0.1049 | -0.0402 | -0.0115 | -0.0384 | -0.0041 | 0.2845 | 0.0702 | -0.0042 |
| 3. ToM 5y. | 0.1364 | 0.2330 | 0.4886 | 0.0513 | 0.2229 | 0.1542 | 0.0000 | 0.0878 | 0.1260 | 0.0457 | 0.1136 | 0.1781 | -0.0442 |
| 4. EF 2y. | 0.0469 | 0.0552 | 0.0513 | 0.2203 | 0.0893 | -0.0364 | -0.0112 | -0.1128 | 0.0365 | -0.0059 | -0.0341 | 0.0446 | -0.0055 |
| 5. Lang 2y. | 0.0811 | 0.1801 | 0.2229 | 0.0893 | 0.7770 | -0.0041 | -0.1234 | -0.0159 | 0.0470 | 0.0308 | 0.0292 | -0.0036 | 0.0701 |
| 6. SES 2y. | 0.1548 | 0.1049 | 0.1542 | -0.0364 | -0.0041 | 0.8767 | 0.0625 | 0.0199 | 0.0672 | -0.0336 | 0.0200 | 0.0710 | 0.0473 |
| 7. No. Sib. 2y. | 0.0307 | -0.0402 | 0.0000 | -0.0112 | -0.1234 | 0.0625 | 0.4100 | -0.1743 | 0.0536 | -0.0217 | -0.0311 | 0.0091 | 0.0321 |
| 8. Prop. Cog. 2y. | -0.1933 | -0.0115 | 0.0878 | -0.1128 | -0.0159 | 0.0199 | -0.1743 | 1.5054 | 0.0794 | 0.1214 | 0.7994 | 0.1069 | -0.0526 |
| 9. Prop. Emo. 2y. | 0.0080 | -0.0384 | 0.1260 | 0.0365 | 0.0470 | 0.0672 | 0.0536 | 0.0794 | 0.3606 | 0.0393 | 0.1414 | 0.1657 | -0.0362 |
| 10. Prop. Des. 2y. | 0.0027 | -0.0041 | 0.0457 | -0.0059 | 0.0308 | -0.0336 | -0.0217 | 0.1214 | 0.0393 | 0.1989 | 0.1517 | 0.0787 | 0.0016 |
| 11. Prop. Cog. 3y. | -0.0198 | 0.2845 | 0.1136 | -0.0341 | 0.0292 | 0.0200 | -0.0311 | 0.7994 | 0.1414 | 0.1517 | 2.2542 | 0.1410 | 0.0979 |
| 12. Prop. Emo. 3y. | 0.0183 | 0.0702 | 0.1781 | 0.0446 | -0.0036 | 0.0710 | 0.0091 | 0.1069 | 0.1657 | 0.0787 | 0.1410 | 0.3885 | -0.0369 |
| 13. Prop. Des. 3y. | -0.0091 | -0.0042 | -0.0442 | -0.0055 | 0.0701 | 0.0473 | 0.0321 | -0.0526 | -0.0362 | 0.0016 | 0.0979 | -0.0369 | 0.1762 |

| **Supplementary Table 6** | | | | | | | | | | | | | | |
| --- | --- | --- | --- | --- | --- | --- | --- | --- | --- | --- | --- | --- | --- | --- |
| *Covariance Matrix for the Vocabulary Size of MST Analyses* | | | | | | | | | | | | | | |
| Variable | 1 | 2 | 3 | 4 | 5 | 6 | 7 | 8 | 9 | 10 | 11 | 12 | 13 |  |
| 1. ToM 3y. | 0.3791 | 0.1462 | 0.1364 | 0.0469 | 0.0811 | 0.1548 | 0.0307 | -0.0635 | -0.0991 | 0.0047 | 0.0491 | 0.0446 | -0.0291 |  |
| 2. ToM 4y. | 0.1462 | 0.6070 | 0.2330 | 0.0552 | 0.1801 | 0.1049 | -0.0402 | -0.2246 | 0.0199 | -0.0712 | 0.3275 | 0.2623 | 0.1304 |  |
| 3. ToM 5y. | 0.1364 | 0.2330 | 0.4886 | 0.0513 | 0.2229 | 0.1542 | 0.0000 | -0.0968 | 0.3608 | 0.0795 | 0.1307 | 0.6591 | 0.0739 |  |
| 4. EF 2y. | 0.0469 | 0.0552 | 0.0513 | 0.2203 | 0.0893 | -0.0364 | -0.0112 | -0.1334 | 0.0566 | 0.0014 | -0.0769 | 0.0503 | -0.0469 |  |
| 5. Lang 2y. | 0.0811 | 0.1801 | 0.2229 | 0.0893 | 0.7770 | -0.0041 | -0.1234 | -0.1608 | 0.1366 | 0.0206 | 0.1413 | 0.3404 | 0.0684 |  |
| 6. SES 2y. | 0.1548 | 0.1049 | 0.1542 | -0.0364 | -0.0041 | 0.8767 | 0.0625 | -0.0137 | 0.0227 | -0.0073 | 0.0475 | 0.2144 | 0.0179 |  |
| 7. No. Sib. 2y. | 0.0307 | -0.0402 | 0.0000 | -0.0112 | -0.1234 | 0.0625 | 0.4100 | -0.1848 | 0.0888 | 0.0293 | -0.1764 | -0.2084 | -0.0247 |  |
| 8. Voc. Cog. 2y. | -0.0635 | -0.2246 | -0.0968 | -0.1334 | -0.1608 | -0.0137 | -0.1848 | 2.3583 | 0.4346 | 0.1874 | 0.0021 | 0.3017 | -0.2783 |  |
| 9. Voc. Emo. 2y. | -0.0991 | 0.0199 | 0.3608 | 0.0566 | 0.1366 | 0.0227 | 0.0888 | 0.4346 | 2.3340 | 0.2998 | 0.2903 | 1.0970 | -0.1829 |  |
| 10. Voc. Des. 2y. | 0.0047 | -0.0712 | 0.0795 | 0.0014 | 0.0206 | -0.0073 | 0.0293 | 0.1874 | 0.2998 | 0.4328 | 0.0435 | 0.1464 | -0.0411 |  |
| 11. Voc. Cog. 3y. | 0.0491 | 0.3275 | 0.1307 | -0.0769 | 0.1413 | 0.0475 | -0.1764 | 0.0021 | 0.2903 | 0.0435 | 2.1733 | 0.6495 | 0.2627 |  |
| 12. Voc. Emo. 3y. | 0.0446 | 0.2623 | 0.6591 | 0.0503 | 0.3404 | 0.2144 | -0.2084 | 0.3017 | 1.0970 | 0.1464 | 0.6495 | 2.7657 | -0.0551 |  |
| 13. Voc. Des. 3y. | -0.0291 | 0.1304 | 0.0739 | -0.0469 | 0.0684 | 0.0179 | -0.0247 | -0.2783 | -0.1829 | -0.0411 | 0.2627 | -0.0551 | 0.8886 |  |

| **Supplementary Table 7** | | | | | | | | | | | |  |
| --- | --- | --- | --- | --- | --- | --- | --- | --- | --- | --- | --- | --- |
| *Estimates for Latent Growth Curve Models of Associations between ToM and Absolute, Proportions, and Vocabulary Range of MST, Excluding Outliers.* | | | | | | | | | | | |  |
|  | Absolute | | |  | Proportions | | |  | Vocabulary | | |  |
| Measure | Est [95% CI] | SE | *p* |  | Est [95% CI] | SE | *p* |  | Est [95% CI] | SE | *p* |  |
|  |  |  |  |  | Regressions with Level in ToM | | |  |  |  |  |  |
| Cog 2 y. | 0.00 [-0.02, 0.01] | 0.01 | 0.708 |  | -0.10 [-0.19, -0.01] | 0.05 | 0.033 |  | -0.01 [-0.09, 0.06] | 0.04 | 0.711 |  |
| Emo 2 y. | -0.01 [-0.04, 0.02] | 0.01 | 0.497 |  | -0.13 [-0.34, 0.08] | 0.11 | 0.231 |  | 0.00 [-0.09, 0.09] | 0.05 | 0.991 |  |
| Des 2 y. | 0.01 [-0.03, 0.05] | 0.02 | 0.511 |  | 0.02 [-0.25, 0.29] | 0.14 | 0.870 |  | -0.03 [-0.22, 0.15] | 0.09 | 0.718 |  |
| Cog 3 y. | 0.00 [-0.01, 0.02] | 0.01 | 0.451 |  | 0.11 [0.01, 0.21] | 0.05 | 0.029 |  | 0.07 [-0.01, 0.14] | 0.04 | 0.091 |  |
| Emo 3 y. | 0.01 [-0.03, 0.05] | 0.02 | 0.625 |  | 0.14 [-0.06, 0.35] | 0.10 | 0.166 |  | 0.08 [-0.01, 0.17] | 0.04 | 0.069 |  |
| Des 3 y. | -0.04 [-0.10, 0.02] | 0.03 | 0.184 |  | -0.44 [-0.75, -0.13] | 0.16 | 0.006 |  | 0.08 [-0.07, 0.22] | 0.07 | 0.308 |  |
| Lang. | 0.22 [0.07, 0.37] | 0.08 | 0.005 |  | 0.23 [0.10, 0.35] | 0.07 | 0.001 |  | 0.15 [0.03, 0.27] | 0.06 | 0.017 |  |
| SES | 0.17 [0.01, 0.33] | 0.08 | 0.040 |  | 0.17 [0.02, 0.31] | 0.07 | 0.024 |  | 0.11 [-0.03, 0.26] | 0.07 | 0.129 |  |
| No. Sib. | 0.01 [-0.17, 0.18] | 0.09 | 0.954 |  | 0.01 [-0.14, 0.17] | 0.08 | 0.866 |  | 0.07 [-0.12, 0.26] | 0.10 | 0.453 |  |
| EF | 0.12 [-0.23, 0.47] | 0.18 | 0.511 |  | 0.05 [-0.26, 0.37] | 0.16 | 0.740 |  | 0.10 [-0.21, 0.40] | 0.16 | 0.547 |  |
|  | Regressions with Change in ToM | | | | | | | | | | |  |
| Cog 2 y. | 0.01 [0.00, 0.03] | 0.01 | 0.035 |  | 0.06 [-0.05, 0.17] | 0.06 | 0.308 |  | -0.02 [-0.09, 0.06] | 0.04 | 0.665 |  |
| Emo 2 y. | -0.01 [-0.05, 0.02] | 0.02 | 0.482 |  | 0.02 [-0.15, 0.20] | 0.09 | 0.810 |  | 0.08 [0.01, 0.16] | 0.04 | 0.027 |  |
| Des 2 y. | -0.01 [-0.05, 0.03] | 0.02 | 0.555 |  | -0.04 [-0.29, 0.21] | 0.13 | 0.757 |  | -0.04 [-0.24, 0.15] | 0.10 | 0.657 |  |
| Cog 3 y. | 0.00 [-0.01, 0.01] | 0.00 | 0.508 |  | 0.02 [-0.05, 0.09] | 0.04 | 0.642 |  | 0.01 [-0.07, 0.09] | 0.04 | 0.833 |  |
| Emo 3 y. | 0.02 [-0.02, 0.06] | 0.02 | 0.383 |  | 0.11 [-0.07, 0.28] | 0.09 | 0.229 |  | 0.07 [-0.02, 0.16] | 0.05 | 0.146 |  |
| Des 3 y. | -0.02 [-0.07, 0.04] | 0.03 | 0.557 |  | -0.20 [-0.56, 0.17] | 0.19 | 0.300 |  | 0.13 [-0.02, 0.28] | 0.07 | 0.079 |  |
| Lang. | 0.07 [-0.07, 0.21] | 0.07 | 0.317 |  | 0.08 [-0.05, 0.21] | 0.07 | 0.244 |  | 0.03 [-0.11, 0.18] | 0.07 | 0.645 |  |
| SES | -0.03 [-0.15, 0.10] | 0.06 | 0.653 |  | -0.03 [-0.14, 0.08] | 0.06 | 0.561 |  | -0.05 [-0.18, 0.07] | 0.06 | 0.382 |  |
| No. Sib. | -0.06 [-0.24, 0.12] | 0.09 | 0.499 |  | -0.05 [-0.22, 0.13] | 0.09 | 0.587 |  | -0.05 [-0.24, 0.14] | 0.10 | 0.606 |  |
| EF | -0.03 [-0.34, 0.27] | 0.15 | 0.831 |  | -0.10 [-0.40, 0.19] | 0.15 | 0.493 |  | -0.15 [-0.41, 0.12] | 0.14 | 0.285 |  |
|  | Latent factors (ToM) | | | | | | | | | | |  |
| Lvl. i. | 1.22 [0.36, 2.08] | 0.44 | 0.005 |  | 1.30 [0.48, 2.13] | 0.42 | 0.002 |  | 1.17 [0.20, 2.14] | 0.50 | 0.019 |  |
| Lvl. v. | 0.11 [0.01, 0.20] | 0.05 | 0.038 |  | 0.07 [-0.01, 0.16] | 0.04 | 0.097 |  | 0.08 [-0.01, 0.16] | 0.04 | 0.080 |  |
| Cha. i. | 0.58 [-0.21, 1.37] | 0.40 | 0.147 |  | 0.66 [-0.02, 1.34] | 0.35 | 0.059 |  | 0.58 [-0.31, 1.46] | 0.45 | 0.203 |  |
| Cha. v. | 0.04 [-0.12, 0.21] | 0.09 | 0.609 |  | 0.01 [-0.15, 0.17] | 0.08 | 0.896 |  | 0.04 [-0.09, 0.16] | 0.06 | 0.571 |  |
| Covar. | 0.00 [-0.10, 0.11] | 0.05 | 0.972 |  | 0.01 [-0.08, 0.10] | 0.05 | 0.824 |  | -0.01 [-0.10, 0.07] | 0.04 | 0.762 |  |
| *Note.* ToM = Theory of mind; MST = Mental state talk; Level = ToM level at 4 years of age; Change = rate of change in ToM level from 3-5 years of age; Cog. = Cognitive; Emo. = Emotion; Des. = Desire; y. = years old; Lang. = Productive language; SES = Socioeconomic status as measured by averaged parental education; No. Sib. = Number of siblings; EF = Executive function; Lvl. = Level; Cha. = Change; i. = intercept; v. = variance; Covar. = Covariance between Level and Change. | | | | | | | | | | | |  |
|  |  |  |  |  |  |  |  |  |  |  |  |  |
|  |  |  |  |  |  |  |  |  |  |  |  |  |
|  |  |  |  |  |  |  |  |  |  |  |  |  |
|  |  |  |  |  |  |  |  |  |  |  |  |  |

| **Supplementary Table 8** | | | | | | | | | | | |  |
| --- | --- | --- | --- | --- | --- | --- | --- | --- | --- | --- | --- | --- |
| *Estimates for Latent Growth Curve Models of Associations between ToM and Absolute, Proportions, and Vocabulary Range of MST, Including Outliers.* | | | | | | | | | | | |  |
|  | Absolute | | |  | Proportions | | |  | Vocabulary | | |  |
| Measure | Est [95% CI] | SE | *p* |  | Est [95% CI] | SE | *p* |  | Est [95% CI] | SE | *p* |  |
|  |  |  |  |  | Regressions with Level of ToM | | |  |  |  |  |  |
| Cog 2 y. | 0.01 [-0.01, 0.02] | 0.01 | 0.310 |  | -0.04 [-0.14, 0.06] | 0.05 | 0.427 |  | -0.01 [-0.08, 0.06] | 0.04 | 0.823 |  |
| Emo 2 y. | -0.01 [-0.04, 0.01] | 0.01 | 0.245 |  | -0.01 [-0.17, 0.16] | 0.09 | 0.935 |  | 0.00 [-0.09, 0.09] | 0.05 | 0.962 |  |
| Des 2 y. | -0.01 [-0.05, 0.03] | 0.02 | 0.649 |  | 0.01 [-0.25, 0.27] | 0.13 | 0.921 |  | -0.04 [-0.23, 0.15] | 0.10 | 0.648 |  |
| Cog 3 y. | 0.00 [-0.01, 0.01] | 0.01 | 0.754 |  | 0.06 [-0.05, 0.18] | 0.06 | 0.276 |  | 0.07 [-0.02, 0.15] | 0.04 | 0.111 |  |
| Emo 3 y. | 0.03 [0.00, 0.06] | 0.02 | 0.063 |  | 0.06 [-0.15, 0.26] | 0.10 | 0.574 |  | 0.09 [0.00, 0.18] | 0.04 | 0.043 |  |
| Des 3 y. | -0.01 [-0.06, 0.04] | 0.02 | 0.625 |  | -0.05 [-0.25, 0.14] | 0.10 | 0.591 |  | 0.08 [-0.07, 0.22] | 0.07 | 0.287 |  |
| Lang. | 0.19 [0.04, 0.34] | 0.08 | 0.014 |  | 0.17 [0.04, 0.31] | 0.07 | 0.014 |  | 0.15 [0.02, 0.27] | 0.06 | 0.020 |  |
| SES | 0.07 [-0.07, 0.20] | 0.07 | 0.345 |  | 0.08 [-0.07, 0.23] | 0.08 | 0.275 |  | 0.05 [-0.07, 0.17] | 0.06 | 0.413 |  |
| No. Sib. | 0.06 [-0.13, 0.25] | 0.10 | 0.531 |  | -0.01 [-0.20, 0.18] | 0.10 | 0.928 |  | 0.10 [-0.10, 0.30] | 0.10 | 0.318 |  |
| EF | 0.15 [-0.21, 0.51] | 0.18 | 0.415 |  | 0.15 [-0.20, 0.50] | 0.18 | 0.406 |  | 0.11 [-0.21, 0.44] | 0.17 | 0.495 |  |
|  |  |  |  |  | Regressions with Change in ToM | | |  |  |  |  |  |
| Cog 2 y. | 0.01 [0.00, 0.03] | 0.01 | 0.019 |  | 0.09 [-0.02, 0.19] | 0.05 | 0.098 |  | -0.01 [-0.07, 0.05] | 0.03 | 0.639 |  |
| Emo 2 y. | -0.02 [-0.05, 0.01] | 0.02 | 0.291 |  | -0.01 [-0.22, 0.19] | 0.11 | 0.908 |  | 0.08 [0.01, 0.16] | 0.04 | 0.030 |  |
| Des 2 y. | -0.03 [-0.07, 0.02] | 0.02 | 0.227 |  | -0.05 [-0.31, 0.20] | 0.13 | 0.682 |  | -0.05 [-0.24, 0.15] | 0.10 | 0.646 |  |
| Cog 3 y. | 0.00 [-0.01, 0.01] | 0.00 | 0.519 |  | 0.00 [-0.08, 0.09] | 0.04 | 0.975 |  | 0.00 [-0.08, 0.08] | 0.04 | 0.950 |  |
| Emo 3 y. | 0.04 [0.01, 0.07] | 0.02 | 0.012 |  | 0.09 [-0.10, 0.27] | 0.09 | 0.361 |  | 0.08 [-0.02, 0.17] | 0.05 | 0.112 |  |
| Des 3 y. | 0.02 [-0.02, 0.06] | 0.02 | 0.311 |  | 0.05 [-0.10, 0.20] | 0.08 | 0.509 |  | 0.14 [0.00, 0.28] | 0.07 | 0.047 |  |
| Lang. | 0.05 [-0.09, 0.18] | 0.07 | 0.512 |  | 0.06 [-0.08, 0.19] | 0.07 | 0.422 |  | 0.03 [-0.11, 0.18] | 0.07 | 0.646 |  |
| SES | -0.11 [-0.19, -0.04] | 0.04 | 0.004 |  | -0.08 [-0.17, 0.00] | 0.04 | 0.057 |  | -0.09 [-0.18,-0.01] | 0.04 | 0.037 |  |
| No. Sib. | -0.01 [-0.18, 0.16] | 0.09 | 0.875 |  | -0.05 [-0.24, 0.14] | 0.10 | 0.582 |  | -0.03 [-0.22, 0.15] | 0.10 | 0.719 |  |
| EF | 0.00 [-0.33, 0.34] | 0.17 | 0.984 |  | 0.00 [-0.31, 0.31] | 0.16 | 0.979 |  | -0.14 [-0.42, 0.13] | 0.14 | 0.304 |  |
|  |  |  |  |  | Latent factors (ToM) | | |  |  |  |  |  |
| Lvl. i. | 1.71 [0.94, 2.47] | 0.39 | 0.000 |  | 1.64 [0.77, 2.51] | 0.44 | 0.000 |  | 1.44 [0.63, 2.25] | 0.41 | 0.000 |  |
| Lvl. v. | 0.08 [-0.04, 0.20] | 0.06 | 0.184 |  | 0.12 [0.03, 0.22] | 0.05 | 0.008 |  | 0.08 [0.00, 0.16] | 0.04 | 0.046 |  |
| Cha. i. | 0.95 [0.41, 1.49] | 0.28 | 0.001 |  | 0.81 [0.24, 1.39] | 0.29 | 0.006 |  | 0.75 [0.10, 1.40] | 0.33 | 0.023 |  |
| Cha. v. | 0.00 [-0.20, 0.20] | 0.10 | 0.991 |  | 0.06 [-0.11, 0.24] | 0.09 | 0.467 |  | 0.03 [-0.09, 0.16] | 0.06 | 0.592 |  |
| Covar. | -0.04 [-0.17, 0.10] | 0.07 | 0.591 |  | 0.03 [-0.08, 0.13] | 0.05 | 0.611 |  | -0.01 [-0.09, 0.08] | 0.04 | 0.858 |  |
| *Note.* ToM = Theory of mind; MST = Mental state talk; Level = ToM level at 4 years of age; Change = rate of change in ToM level from 3-5 years of age; Cog. = Cognitive; Emo. = Emotion; Des. = Desire; y. = years old; Lang. = Productive language; SES = Socioeconomic status as measured by averaged parental education; No. Sib. = Number of siblings; EF = Executive function; Lvl. = Level; Cha. = Change; i. = intercept; v. = variance; Covar. = Covariance between Level and Change. | | | | | | | | | | | |  |
|  |  |  |  |  |  |  |  |  |  |  |  |  |
|  |  |  |  |  |  |  |  |  |  |  |  |  |
|  |  |  |  |  |  |  |  |  |  |  |  |  |
|  |  |  |  |  |  |  |  |  |  |  |  |  |
