# Supplementary material for: The longitudinal relations between mental state talk and theory of mind
Source: BMC Psychol. 2024 Apr 6;12:191. doi: 10.1186/s40359-024-01692-y (PMC10998333; doi:10.1186/s40359-024-01692-y)
Supplement: Supplementary file 4 — Supplementary Material 4. [file 40359_2024_1692_MOESM4_ESM.docx]

| **Supplementary Table 9** | |
| --- | --- |
| *Observed Guttman Scale Patterns in the Current Data.* | |
| Pattern | Count |
| *0000* | *1* |
| *0001* | *12* |
| 0010 | 3 |
| *0011* | *75* |
| 0100 | 1 |
| 0101 | 5 |
| 0110 | 2 |
| *0111* | *59* |
| 1000 | 0 |
| 1001 | 0 |
| 1010 | 0 |
| 1011 | 4 |
| 1100 | 1 |
| 1101 | 0 |
| 1110 | 0 |
| *1111* | *30* |
| Note. Core patterns are italicized, and other patterns are error patterns. The numbers in the first column present the pattern in reverse order of difficulty. 0001 means that the child succeeded on the DD step, but no other steps. 1000 means that the child succeeded on the CFB step, but no other steps. | |

| **Supplementary Table 10** |  |  |  |  |
| --- | --- | --- | --- | --- |
| *Values Extracted (in Accordance with Green's Method) from the Current Data for Calculation of Guttman Scalogram Analyses, specifically Reproducibility (Rep) and Index of consistency (I).* | | | | |
| ToM Scale Step | CFB | KA | DB | DD |
| FormS2 (correct) | 35 | 98 | 173 | 185 |
| Form6 (incorrect) | 158 | 95 | 20 | 8 |
| Form4 (1, g) | NA | 4 | 7 | 5 |
| Form5a (1,1,g,0) | NA | NA | 1 |  |
| Form5b (1, ,g) | NA | NA | 1 | 4 |
| Note. CFB = Content false belief; KA = Knowledge Acquisition; DB = Diverse Belief; DD = Diverse Desires; FormS2 is the sum of correct responses across the sample. Form6 is calculated by using the formula (FormS2-number of participants). Form4 is the number of times a participant is failed on the current task (g) but was successful on the next (more difficult) task (g+1). Form5a is the number of times a participant fails the first (in this case also easiest) and second task (g), but is successful on the two following (g+1 and g+2) following. Form5b calculates the number of times a participant fails the current task (g) and is successful on the task after the next task (g+2). | | | | |

| **Supplementary Table 11** |  |
| --- | --- |
| *Guttman Scalogram Results for the Current Data.* | |
| Guttman measure | Value |
| Rep^a^ | 0.9779793 |
| Rep^b^ | 0.9792478 |
| Rep^i^ | 0.9551415 |
| *I* ^a^ | 0.5091067 |
| *I* ^b^ | 0.5373843 |
| *Note*. Rep = Reproducibility; *I* = Index of consistency; *I* ^a^ is calculated using the formula (Rep^a^-Rep^i^) / (1-Rep^i^). *I* ^b^ is calculated using the formula (Rep^b^-Rep^i^) / (1-Rep^i^). | |
